# Effect of Prewarming during Induction of Anesthesia on Microvascular Reactivity in Patients Undergoing Off-Pump Coronary Artery Bypass Surgery: A Randomized Clinical Trial

**DOI:** 10.1371/journal.pone.0159772

**Published:** 2016-07-21

**Authors:** Youn Joung Cho, Seo Yun Lee, Tae Kyong Kim, Deok Man Hong, Yunseok Jeon

**Affiliations:** Department of Anesthesiology and Pain Medicine, Seoul National University Hospital, Seoul, South Korea; Campus Bio-Medico University, ITALY

## Abstract

**Background:**

General anesthesia may induce inadvertent hypothermia and this may be related to perioperative cardiovascular complications. Microvascular reactivity, measured by the recovery slope during a vascular occlusion test, is decreased during surgery and is also related to postoperative clinical outcomes. We hypothesized that microvascular changes during surgery may be related to intraoperative hypothermia. To evaluate this, we conducted a randomized study in patients undergoing off-pump coronary artery bypass surgery, in which the effect of prewarming on microvascular reactivity was evaluated.

**Methods:**

Patients scheduled for off-pump coronary artery bypass surgery were screened. Enrolled patients were randomized to the prewarming group to receive forced-air warming during induction of anesthesia or to the control group. Measurement of core and skin temperatures and vascular occlusion test were conducted before anesthesia induction, 1, 2, and 3 h after induction, and at the end of surgery.

**Results:**

In total, 40 patients were enrolled and finished the study (*n* = 20 in the prewarming group and *n* = 20 in the control group). During the first 3 h of anesthesia, core temperature was higher in the prewarming group than the control group (*p* < 0.001). The number of patients developing hypothermia was lower in the prewarming group than the control group (4/20 vs. 13/20, *p* = 0.004). However, tissue oxygen saturation and changes in recovery slope following a vascular occlusion test at 3 h after anesthesia induction did not differ between the groups. There was no difference in clinical outcome, including perioperative transfusion, wound infection, or hospital stay, between the groups.

**Conclusions:**

Prewarming during induction of anesthesia decreased intraoperative hypothermia, but did not reduce the deterioration in microvascular reactivity in patients undergoing off-pump coronary artery bypass surgery.

**Trial Registration:**

ClinicalTrials.gov NCT02186210

## Introduction

During general anesthesia, core temperature (T_core_) declines even in actively warmed patients [1]. Anesthesia-induced hypothermia is related to altered thermoregulation [2], exposure to a cold environment, and redistribution of body heat [3]. Inadvertent intraoperative hypothermia (defined as T_core_ < 36°C) increases perioperative adverse cardiac events,[4] perioperative transfusion [1, 5], coagulopathy [6], wound infection [7, 8], and prolonged hospitalization [1, 7].

Tissue oxygen saturation (StO_2_) and StO_2_-derived dynamic parameters during a vascular occlusion test (VOT) reflect tissue microcirculation and microvascular reactivity with local reperfusion reserve [9, 10]. StO_2_ and its parameters have been shown to decrease in septic or cardiac surgical patients [11–13], and these alterations were related to poor clinical outcomes [13–17].

In previous investigations, peripheral microcirculation and microvascular reactivity were affected markedly during therapeutic hypothermia in newborns [18] and cardiac arrest patients [19]. However, the influence of intraoperative hypothermia on tissue microperfusion has not been investigated in cardiac surgical patients. We hypothesized that prewarming during the period of induction of anesthesia would attenuate intraoperative hypothermia and may enhance microcirculation in patients undergoing off-pump coronary artery bypass (OPCAB) surgery.

## Methods

This study was approved by the Institutional Review Board of Seoul National University Hospital (ref # 1306-026-496; S1 File), and was registered at ClinicalTrials.gov (identifier, NCT02186210; S2 File). All patients gave written informed consent. The study was conducted in accordance with Good Clinical Practice guidelines and the principles of the Declaration of Helsinki.

### Patient selection

**Fig 1:**
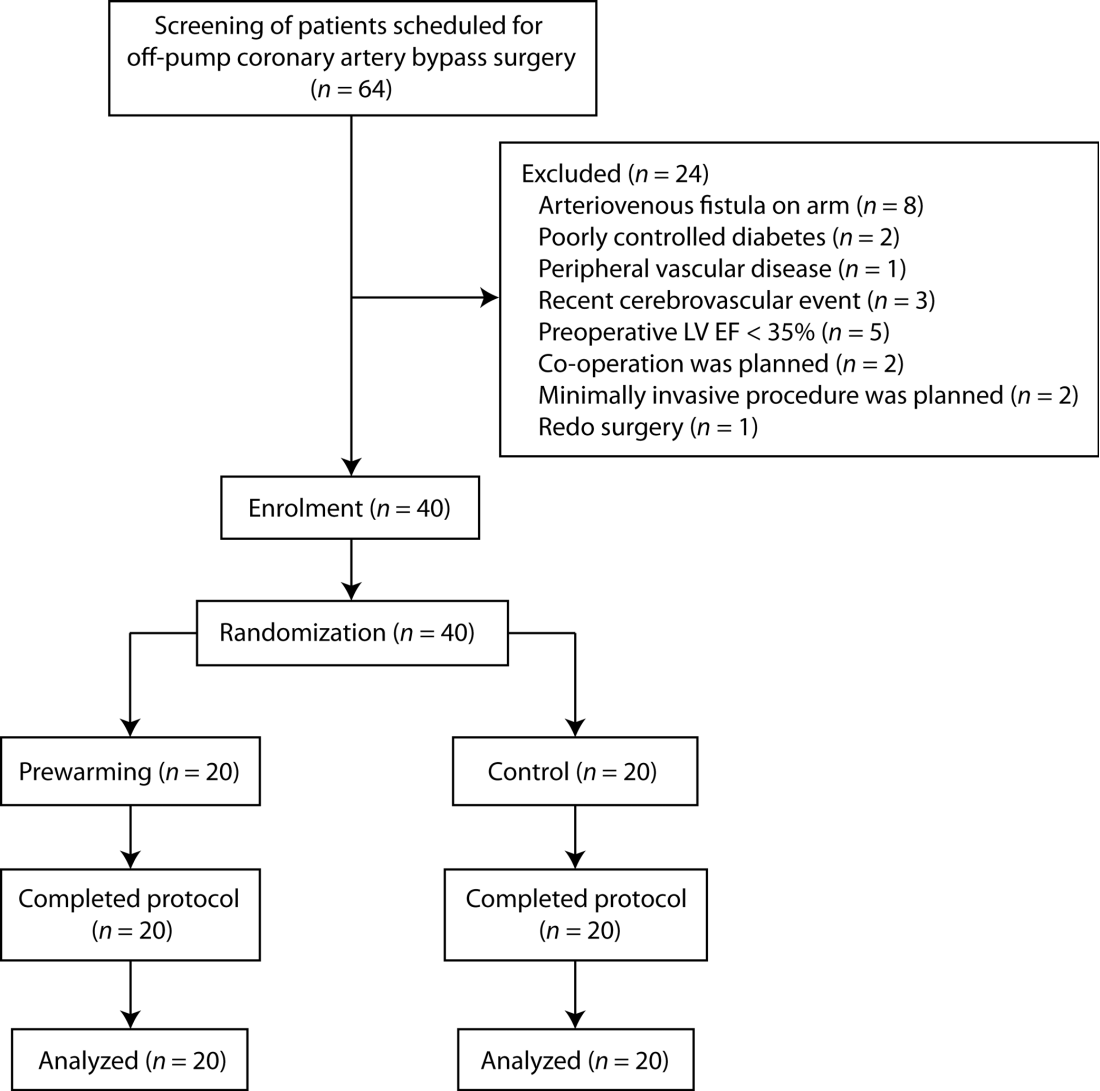
CONSORT flow diagram.

Patients scheduled for OPCAB surgery at Seoul National University Hospital were screened for eligibility. Exclusion criteria were age < 20 or > 85 years, presence of anatomical anomaly or arteriovenous fistula in the upper extremities, peripheral vascular disease or recent cerebrovascular event (within 6 months), diabetes requiring insulin therapy, preoperative left ventricular (LV) ejection fraction (EF) < 35%, requirement of preoperative inotropes or ventricular assist devices, redo or combined surgery, other than a conventional operative procedure (such as a minimally invasive approach), and pregnancy (Fig 1).

After providing informed consent, patients were randomized 1:1 to the prewarming or control group. Block randomization (blocks of 4) was executed using a computer-generated program by an independent clinician and allocation concealment was kept until data analyses. Baseline patient data collection and laboratory tests including arterial blood gas analysis were performed before surgery. The operative risk was assessed and calculated using the European System for Cardiac Operative Risk Evaluation (EuroSCORE II) [20].

### Study protocol

Without premedication, patients were monitored with five-lead electrocardiography, pulse oximetry, non-invasive blood pressure (BP), bispectral index, and cerebral oximetry using a non-infrared spectroscopic sensor in the operating room. After local analgesia with 1% lidocaine injection, the right or left radial artery was cannulated with a 20-G Angiocath™ (Becton Dickinson Medical Ltd., Tuas, Singapore) and connected to a FloTrac™ transducer and EV1000™ monitor (Edwards Lifesciences LLC, Irvine, CA, USA). From them, arterial BP, cardiac index (CI), and stroke volume variation were monitored continuously.

Anesthesia was induced with intravenous injection of midazolam 1.5 mg/kg, vecuronium 1.5 mg/kg, and sufentanil 1 μg/kg. After tracheal intubation, the lungs were ventilated with volume-controlled mechanical ventilation with a tidal volume of 7–8 mL/kg, fraction of inspired oxygen of 0.5, and a respiratory rate of 9–12/min, to maintain the arterial partial pressure of carbon dioxide at 35–45 mmHg without positive end-expiratory pressure. After placement of a central venous catheter (AVA HF; Edwards Lifesciences LLC) in the right or left internal jugular vein, a pulmonary artery (PA) catheter (Swan-Ganz CCOmbo V, model 774HF75; Edwards Lifesciences LLC) was inserted through the internal jugular lumen and was connected to a Vigilance™ II monitor (Edwards Lifesciences LLC) with *in vivo* calibration. CI and mixed venous saturation (SvO_2_) were monitored through the Vigilance™ II monitor after placing the PA catheter.

Anesthesia was maintained with continuous infusion of propofol (Fresofol®2 MCT 2%; Fresenius Kabi, Graz, Austria) and remifentanil (Ultiva™; GlaxoSmithKline, San Polo di-Torrile, Italy) in an effect site target-controlled infusion (TCI) mode. For TCI, the effect site concentration (Ce) was set to 1–4 μg/mL for propofol and 5–12 ng/mL for remifentanil using a commercial infusion pump (Orchestra®; Fresenius Vial, Brezins, France). Ce was monitored with the Marsh pharmacokinetic model for propofol and the Minto model for remifentanil. Anesthetic depth was monitored using an electroencephalogram-based monitor (BIS VISTA™ monitor; Aspect Medical Systems, Norwood, MA, USA) to maintain a bispectral index between 30 and 50. Crystalloid was used for fluid maintenance and blood products were transfused according to the hemodynamic and laboratory variables. According to the center protocol, red cell transfusion was initiated considering hematocrit (to maintain > 27–30%), ongoing surgical bleeding, hemodynamic parameters, and underlying comorbidities. Administration of fluids and vasoactive agents was managed according to the attending anesthesiologist’s decision to maintain mean BP > 60 mmHg, CI > 2.0 L/min/m^2^, and SvO_2_ > 65%, according to the institutional practice. The primary choice of inotropic agent was noradrenalin in most cases. Intravenous infusates were administered using a fluid warming device (Level 1; Smiths Industries Medical System, Rockland, MA, USA) in both groups. Total intravenous anesthesia was maintained until the end of surgery and discontinued thereafter. All patients were transferred to a cardiopulmonary intensive care unit (ICU) after the surgery.

Before induction of anesthesia, T_core_ was measured at the tympanic membrane (T_tymp_) using a tympanic thermometer (Thermoscan® IRT4520; Braun GmbH, Kronberg, Germany). The right and left T_tymp_ were measured, both twice, and the average of the measured temperatures was recorded. After insertion of a PA catheter, T_core_ was monitored through the PA catheter. Two skin surface temperatures were monitored with skin temperature probes placed at the tip of the index finger and on the forearm. From them, temperature of finger (T_finger_) and forearm (T_forearm_) were monitored, and the differences between T_core_ or T_forearm_ and T_finger_ (ΔT_core-finger_ and ΔT_forearm-finger_) were recorded.

Patients in the prewarming group were heated using a forced-air warmer (Bair Hugger™ Model 505; 3M, St. Paul, MN, USA) connected to a full body blanket (Bair Hugger™ Model 30000; Arizant Healthcare Inc., Eden Prairie, MN, USA), also covered with a cotton blanket, with the air heating temperature set at 43°C. Patients were covered from the upper trunk to the lower leg except an arm for monitoring of skin temperatures. Prewarming was performed, after the placement of monitoring devices, until the end of induction of anesthesia. Patients in the control group were covered with a single cotton blanket without additional air heating. All patients used an automated coil-heated mattress (Blanketrol® II; Cincinnati Sub-Zero Products Inc., Cincinnati, OH, USA) placed on the operative table, set at 37°C.

T_core_, T_finger_, and T_forearm_ were recorded before the induction of anesthesia, and at 1, 2, and 3 h after induction of anesthesia, and at the end of surgery. The temperature of the operating room (T_OR_) was kept between 20 and 23°C, automatically controlled by an operating room control system, and was recorded at the same time points. After the first 3 h post-induction, if the patients’ T_core_ reached 36.9°C, or above 36.7°C with a continuously increasing pattern with ≥ 0.1°C increase for three consecutive 10-min intervals, body surface cooling was applied using the automated mattress (temperature set at 23°C) until the T_core_ reached 36.5°C. Then, the mattress temperature was returned to the initial setting.

**Fig 2:**
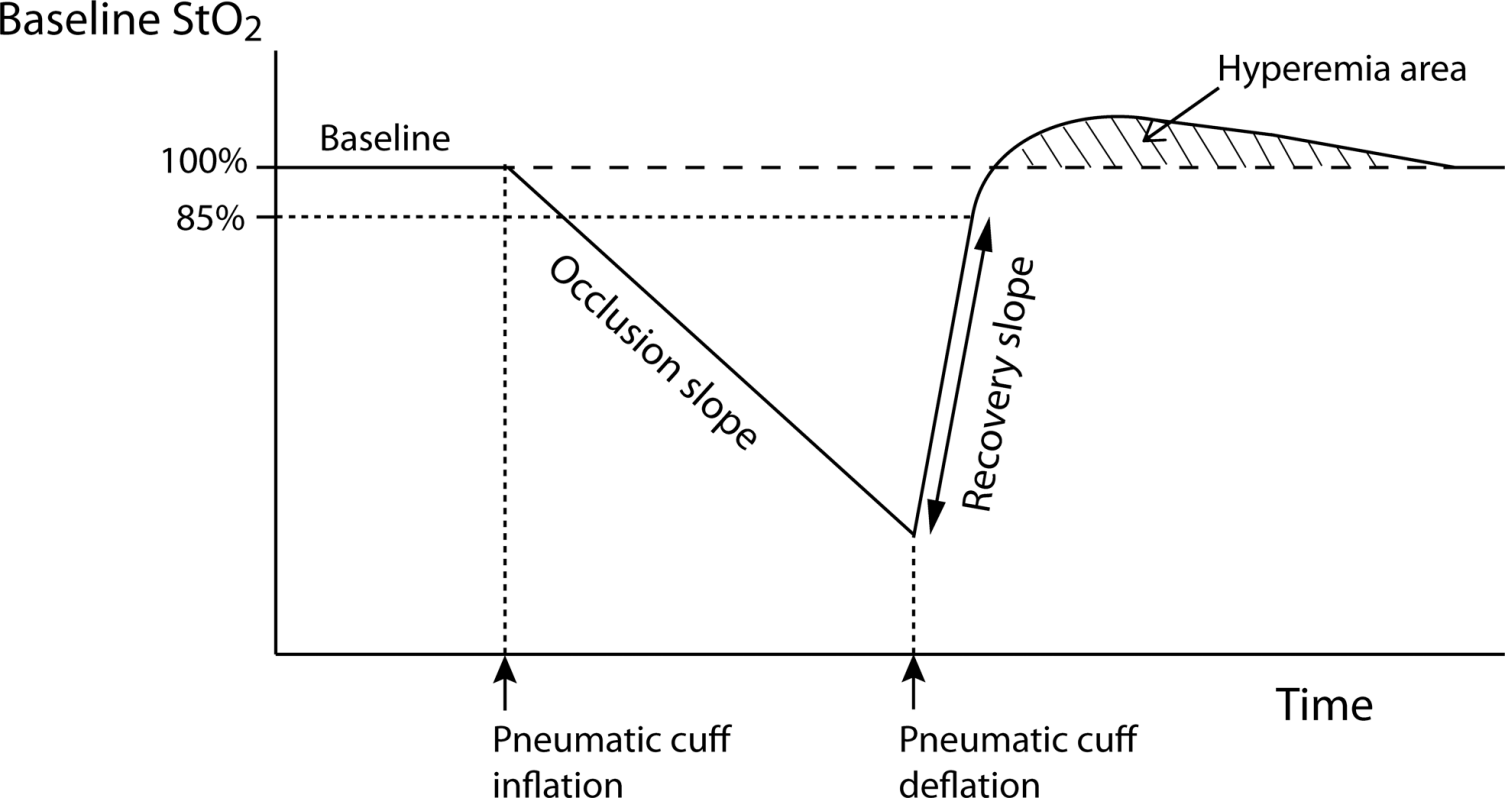
Changes in tissue oxygen saturation during the vascular occlusion test. StO_2_, tissue oxygen saturation.

StO_2_ and StO_2_-derived parameters during VOT were monitored with an Inspectra™ StO_2_ Tissue Oxygenation Monitor (model 650; Hutchinson Technology Inc., Hutchinson, MN, USA) with an Inspectra™ StO_2_ Sensor (model 1615; Hutchinson Technology Inc.) attached to the thenar eminence of the hand of the opposite side for monitoring of T_finger_ and T_forearm_. VOT was performed by inflating the pneumatic cuff placed at the upper arm up to 50 mmHg above the systolic BP. The cuff remained inflated until the StO_2_ reached 40% (occlusion of vessel), and was then deflated rapidly. The occlusion slope was defined as the slope during 1 min after initiating cuff inflation. The recovery slope was calculated from the deflation of the cuff until the restoration of 85% of the baseline (Fig 2). VOT was performed before the induction of anesthesia, and at 1, 2, and 3 h after induction of anesthesia, and at the end of surgery. An experienced researcher (YJC), blinded to the randomization, analyzed the VOT graphs using the Inspectra Analysis Program (ver. 4.03; Hutchinson Technology Inc.) and recorded the VOT parameters.

Arterial blood gas analysis was performed at the same time points as the temperature recordings. Cardiac troponin I levels were measured before surgery, on ICU admission, 6 h after surgery, and on postoperative days 1, 2, 3, 5, and 7.

### Statistical analyses

The primary endpoint was difference in recovery slope during VOT at 3 h after induction of anesthesia. We considered that a 50% between-group difference would have clinical relevance in the current study protocol. We calculated the number of patients using the data from the heart surgery registry at Seoul National University Hospital (ClinicalTrials.gov identifier, NCT01713192): decrease in recovery slope at 3 h after induction of anesthesia was 1.5 ± 0.7%/s (mean ± SD). From the results, we calculated the number of patients required to be 20 per group to detect a 50% decrease in recovery slope at 3 h after induction, relative to baseline, with a two-sided design at a significance level of 5% and 90% power. The G*Power software package (ver. 3.1.9.2; Franz Faul, Universitat Kiel, Germany) was used for the power calculation.

Data are presented as means ± SD or as numbers (%). Data were compared using Student’s *t*-test, the Mann-Whitney U-test, Pearson’s χ^2^ test, or Fisher’s exact test. Serial changes in values were analyzed using linear mixed models. In the mixed model, the treatment group, time, and the interaction between group and time were regarded as fixed effects, and subject was regarded as a random effect. Residuals versus fitted values plots were used to check that the error terms (residuals) had a mean of zero and constant variance. The plots did not show any pattern against the equal variance assumption. The normality assumption for the model residuals was checked with histograms and normal quantile-quantile (Q-Q) plots of residuals, which seemed to be normally distributed. *P* values were adjusted using the Hochberg method to correct for multiple testing. Analyses were performed using SPSS for Windows software (ver. 21.0.0.0; IBM, Armonk, NY, USA). A *p* value < 0.05 was taken to indicate statistical significance.

## Results

Patient enrolment and data collection were performed between August 2014 and February 2015. Among the 64 patients screened for eligibility, 24 were excluded: 8 of these patients had arteriovenous fistulas for hemodialysis in their arms, 2 had poorly controlled diabetes requiring insulin injection, 1 had peripheral vascular obstructive disease, 3 had histories of cerebrovascular events within 6 months, preoperative LV EF was < 35% in 5 patients, co-operations for other organs were planned in 2 patients, minimally invasive procedures were planned in 2 patients, and 1 was a redo surgery. In total, 40 patients were randomized to the prewarming (*n* = 20) or control group (*n* = 20). All included patients completed the protocol and were analyzed (Fig 1 and S1 Fig).

Patient baseline and perioperative characteristics, including target vessels for coronary revascularization and main procedural (anastomosis) time, were similar between the groups (Table 1). The mean warming time in the prewarming group was 35 ± 6 min. There was no difference in the temperatures (T_OR_, T_finger_, T_forearm_, ΔT_core-finger_, and ΔT_forearm-finger_) during surgery between the two groups except for T_core_ (Table 2). T_core_ was higher in the prewarming group than the control group during the first 3 h after induction (all adjusted *p* < 0.001, Table 2). At the end of surgery, T_core_ was comparable between the groups (adjusted *p* = 0.464, Table 2). The interaction between the time and group was significant for T_core_ (*p* < 0.001). The number of patients who developed intraoperative hypothermia (T_core_ < 36.0°C at any time during the surgery) was lower in the prewarming group (4/20 [20%] vs. 13/20 [65%], *p* = 0.004). The nadir T_core_ during surgery was 36.2 ± 0.3°C vs. 35.7 ± 0.2°C in the prewarming vs. control groups, respectively (*p* < 0.001). Body surface cooling was applied intraoperatively after 3 h after induction of anesthesia in 14/20 (70%) and 8/20 (40%) patients in the prewarming and control groups, respectively (*p* = 0.057). Additional forced-air warming was required intraoperatively in one patient in the control group, but in no patient in the prewarming group (*p* > 0.999). On ICU admission immediately after surgery, axillary temperature was 36.6 ± 0.4°C vs. 36.5 ± 0.4°C in the prewarming vs. control groups, respectively (*p* = 0.261).

**Table 1:** Baseline and perioperative patients’ characteristics.

		Prewarming group	Control group	*p*-value
		(*n* = 20)	(*n* = 20)	
Age (yr)		63 ± 9	66 ± 8	0.281
Female		6 (30%)	4 (20%)	0.716
Body mass index (kg/m^2^)		24.6 ± 2.2	24.8 ± 3.4	0.758
Body surface area (m^2^)		1.7 ± 0.2	1.7 ± 0.2	0.612
ASA class				0.342
	II	4 (20%)	1 (5%)	
	III	16 (80%)	19 (95%)	
Smoking history				0.546
	Never smoker	8 (40%)	9 (45%)	
	Current smoker	7 (35%)	4 (20%)	
	Ex-smoker	5 (25%)	7 (35%)	
Co-morbidities				
	Hypertension	14 (70%)	11 (55%)	0.327
	Diabetes	6 (30%)	12 (60%)	0.057
	Cerebrovascular disease	1 (5%)	4 (20%)	0.342
	Dyslipidemia	10 (50%)	11 (55%)	0.752
	Chronic kidney dysfunction	0 (0%)	0 (0%)	
Preoperative medications				
	ACEi	6 (30%)	3 (15%)	0.451
	ARB	5 (25%)	2 (10%)	0.407
	BB	10 (50%)	11 (55%)	0.752
	CCB	12 (60%)	8 (40%)	0.206
	Diuretics	0 (0%)	1 (5%)	> 0.999
	Aspirin	19 (95%)	18 (90%)	> 0.999
	Clopidogrel	1 (5%)	0 (0%)	> 0.999
	Statins	17 (85%)	15 (75%)	0.695
	OHA	6 (30%)	9 (45%)	0.327
	Nitrate	15 (75%)	15 (75%)	> 0.999
	Nitroglycerine	20 (100%)	20 (100%)	
	Nicorandil	6 (30%)	5 (25%)	0.723
Preoperative LV EF (%)		60 ± 6	56 ± 7	0.118
Preoperative hemoglobin (g/dL)		13.4 ± 1.2	12.8 ± 1.5	0.166
Preoperative troponin I (ng/mL)		0.08 ± 0.24	0.06 ± 0.19	0.793
EuroSCORE II (%)		1.3 ± 0.9	1.5 ± 0.8	0.298
Number of grafts		4 (2–4 [1–5])	4 (3–4 [1–6])	0.516
Target vessels				
	LAD	17 (85%)	18 (90%)	> 0.999
	D	14 (70%)	17 (85%)	0.451
	OM	13 (65%)	16 (80%)	0.288
	PDA	13 (65%)	9 (45%)	0.204
	PLB	5 (25%)	4 (20%)	> 0.999
	R or RI	4 (20%)	3 (15%)	> 0.999
	RCA	0 (0%)	2 (10%)	0.487
Anesthesia time (min)		437 ± 50	435 ± 68	0.917
Coronary anastomosis time (min)		105 ± 34	109 ± 41	0.735
Infused crystalloid (mL)		3335 ± 1253	3998 ± 1519	0.140
Infused colloid (mL)		0	0	
Estimated blood loss (mL)		901 ± 374	1013 ± 513	0.438
Intraoperative urinary output (mL)		1140 ± 605	1276 ± 894	0.576

Values are means ± SD, numbers (%) or median (IQR [range]). ACEi, angiotensin-converting enzyme inhibitor; ARB, angiotensin receptor blocker; BB, beta-blocker; CCB, calcium channel blocker; OHA, oral hypoglycemic agent; LV, left ventricular; EF, ejection fraction; LAD, left anterior descending artery; D, diagonal branch; OM, obtuse marginal branch; PDA, posterior descending artery; PLB, posterolateral branch; R, ramus branch; RI, ramus intermedius branch; RCA, right coronary artery.

**Table 2:** Changes in temperatures, microcirculatory parameters and use of vasoactive agents of the prewarming and control group during off-pump coronary artery bypass surgery.

	Group	Baseline	1 H	2 H	3 H	End of surgery
T_OR_ (°C)	P	21.7 ± 1.6	22.2 ± 1.5	22.3 ± 1.6	22.5 ± 1.7	22.7 ± 1.5
	C	21.4 ± 1.8	21.9 ± 1.7	22.0 ± 1.8	22.1 ± 1.8	22.5 ± 1.7
T_core_ (°C)	P	36.6 ± 0.3	36.5 ± 0.3*	36.2 ± 0.3*	36.4 ± 0.4*	36.7 ± 0.3
	C	36.7 ± 0.2	36.0 ± 0.3*	35.7 ± 0.4*	35.9 ± 0.4*	36.6 ± 0.4
T_finger_ (°C)	P	29.7 ± 3.2	34.0 ± 2.5	34.5 ± 2.0	34.9 ± 1.9	32.9 ± 3.0
	C	29.4 ± 2.6	33.7 ± 1.7	34.0 ± 1.6	34.3 ± 1.5	33.7 ± 1.7
T_forearm_ (°C)	P	31.9 ± 0.9	34.2 ± 1.1	35.4 ± 0.6	35.8 ± 0.6	35.6 ± 0.8
	C	31.7 ± 1.4	33.5 ± 1.5	34.6 ± 0.8	35.1 ± 0.7	35.6 ± 0.5
ΔT_core-finger_ (°C)	P	6.9 ± 3.3	2.4 ± 2.5	1.8 ± 1.9	1.5 ± 1.8	3.8 ± 3.1
	C	7.3 ± 2.6	2.3 ± 1.8	1.7 ± 1.6	1.5 ± 1.5	2.9 ± 1.7
ΔT_forearm-finger_ (°C)	P	2.2 ± 2.9	0.2 ± 2.1	1.0 ± 1.6	0.8 ± 1.4	2.7 ± 2.7
	C	2.3 ± 2.4	–0.2 ± 1.6	0.6 ± 1.2	0.8 ± 1.1	1.9 ± 1.5
StO_2_ (%)	P	81 ± 7	81 ± 8	75 ± 10	76 ± 10	71 ± 10
	C	82 ± 7	82 ± 4	78 ± 6	79 ± 7	71 ± 9
Occlusion slope (%/min)	P	–7.5 ± 1.8	–7.0 ± 2.7	–7.7 ± 2.7	–8.0 ± 1.6	–8.2 ± 2.0
	C	–7.9 ± 2.0	–7.5 ± 1.5	–7.9 ± 1.3	–7.8 ± 1.9	–9.3 ± 1.7
Recovery slope (%/s)	P	3.4 ±1.4	2.9 ± 1.3	2.9 ± 1.3	2.8 ± 1.0	2.7 ± 0.8
	C	3.6 ± 1.2	3.5 ± 1.2	3.3 ± 1.3	3.1 ±1.5	2.8 ± 1.1
Use of vasoactive agents**						
Phenylephrine (μg)	P	N/A	8 ± 16	43 ± 56	8 ± 16	183 ± 183
	C	N/A	4 ± 12	35 ± 69	17 ± 37	183 ± 239
Vasopressin (unit)	P	N/A	0.01 ± 0.02	0.01 ± 0.04	0.01 ± 0.02	0.27 ± 0.35
	C	N/A	0	0.02 ± 0.07	0.02 ± 0.07	0.56 ± 0.93
Continuous infusion of noradrenalin						
Number of patients	P	N/A	1 (5%)	7 (35%)	8 (40%)	16 (80%)
	C	N/A	2 (10%)	6 (30%)	7 (35%)	16 (80%)
Max dose (μg/kg/min)	P	N/A	0.001 ± 0.003	0.007 ± 0.010	0.009 ± 0.012	0.020 ± 0.020
	C	N/A	0.001 ± 0.003	0.004 ± 0.007	0.006 ± 0.010	0.020 ± 0.010
Duration (min)	P	N/A	1 ± 3	13 ± 21	21 ± 28	138 ± 130
	C	N/A	1 ± 5	11 ± 20	18 ± 26	158 ± 137

Values are means ± SD or numbers (%). Interaction between time and group was significant for T_core_ (*p* < 0.001); and not significant for T_finger_ (*p* = 0.364), T_forearm_ (*p* = 0.057), ΔT_core-finger_ (*p* = 0.550), ΔT_forearm-finger_ (*p* = 0.739), StO_2_ (*p* = 0.615), occlusion slope (*p* = 0.415), or recovery slope (*p* = 0.834).

1 H, 1 h after induction of general anesthesia; 2 H, 2 h after induction of general anesthesia; 3 H, 3 h after induction of general anesthesia; T_OR_, temperature of operating room; T_core_, core temperature; P, prewarming group; C, control group; T_finger_, temperature of fingertip; T_forearm_, temperature of forearm; ΔT_core-finger_, difference of T_core_ and T_finger_; ΔT_forearm-finger_, difference of T_forearm_ and T_finger_; StO_2_, tissue oxygen saturation; N/A, not assessed.

* Adjusted *p* < 0.001 between the two groups.

** Dose of vasoactive agents at each time point indicates consecutive dose until 3 h (1 H, from induction to 1 h after induction; 2 H, from 1 h to 2 h after induction; 3 H, from 2 h to 3 h after induction), and total cumulative dose at the end of surgery.

Skin temperature measured at the finger was not different between the groups (*p* = 0.751), whereas temperatures at the forearm were higher in the prewarming group than the control group during the surgery (mean difference, 0.5°C [95% CI 0.0–0.9°C]; *p* = 0.037; Table 2). The interactions between time and group were not significant (*p* = 0.364 and *p* = 0.057 for T_finger_ and T_forearm_, respectively). However, ΔT_core-finger_ and ΔT_forearm-finger_ were similar between the groups (*p* = 0.800 and *p* = 0.478, respectively; Table 2). The interactions between time and group were not significant (*p* = 0.550 and *p* = 0.739 for ΔT_core-finger_ and ΔT_forearm-finger_, respectively).

The primary endpoint, decrease in recovery slope at 3 h after induction of anesthesia, did not differ between the groups (–0.6 ± 1.6 vs. –0.5 ± 2.0%/s in the prewarming vs. control groups, respectively, *p* = 0.953). Throughout the surgery, there was no difference in occlusion or in recovery slope between the groups (*p* = 0.367 and *p* = 0.229, respectively; Table 2). StO_2_ values were similar between the groups during surgery (*p* = 0.516, Table 2). The interactions between time and group were not significant (*p* = 0.615, *p* = 0.415 and *p* = 0.834, for StO_2_, occlusion and recovery slope, respectively).

Other hemodynamic and laboratory variables were similar between the groups except for SvO_2_ (Table 3). For repeated measured values, SvO_2_ was higher in the prewarming group than the control group (*p* = 0.040; mean difference, 3% [95% CI 0–6%]), although baseline values could not be obtained (Table 3). Intraoperative use of vasoactive agents, such as phenylephrine, vasopressin, and noradrenalin was comparable between the two groups (Table 2). None of the patients required adrenalin intraoperatively. The levels of postoperative troponin I until postoperative day 7 were also comparable between the groups. There was one mortality case in the control group. The patient developed cerebral infarction in the territory of the left middle cerebral artery on the first postoperative day. The patient underwent an immediate craniectomy, but developed multiple organ ischemic damage and eventually had cardiac arrest, which was not resuscitated, on the 39th postoperative day. There was no difference in the amount of perioperative transfusion, chest tube drain, time to extubate, postoperative complications including wound infection, or ICU or hospital stay between the groups (Table 4). At the 1-year follow-up after surgery, more than 94% of grafts were patent by computed tomographic or percutaneous coronary angiography in both groups; there was no difference between the groups (Table 4).

**Table 3:** Hemodynamic and laboratory variables of the prewarming and control group during off-pump coronary artery bypass surgery.

	Group	Baseline	1 H	2 H	3 H	End of surgery
HR (beats/min)	P	65 ± 8	58 ± 8	64 ± 13	64 ± 12	79 ± 13
	C	68 ± 8	56 ± 9	58 ± 9	56 ± 11	79 ± 14
MBP (mmHg)	P	103 ± 19	72 ± 10	71 ± 8	74 ± 9	74 ± 9
	C	103 ± 19	72 ± 9	74 ± 7	78 ± 11	72 ± 7
MPAP (mmHg)	P	N/A	13 ± 3	16 ± 2	16 ± 3	16 ± 4
	C	N/A	13 ± 4	16 ± 4	15 ± 4	15 ± 5
CI (L/min/m^2^)	P	N/A	2.1 ± 0.4	2.2 ± 0.5	2.2 ± 0.5	2.3 ± 0.4
	C	N/A	2.2 ± 0.8	2.0 ± 0.3	2.1 ± 0.4	2.2 ± 0.4
SVV (%)	P	10 ± 5	10 ± 4	10 ± 3	7 ± 3	10 ± 3
	C	7 ± 3	10 ± 3	9 ± 4	7 ± 2	12 ± 4
Lactate (mmol/L)	P	0.9 ± 0.4	0.9 ± 0.4	0.9 ± 0.3	0.8 ± 0.2	0.9 ± 0.3
	C	0.9 ± 0.3	1.0 ± 0.4	1.0 ± 0.4	0.9 ± 0.4	1.0 ± 0.3
pH	P	7.43 ± 0.03	7.44 ± 0.03	7.42 ± 0.04	7.42 ± 0.04	7.41 ± 0.04
	C	7.43 ± 0.03	7.43 ± 0.05	7.42 ± 0.05	7.42 ± 0.06	7.40 ±0.05
Base deficit (mmol/L)	P	-1.8 ± 2.3	-1.5 ± 2.1	-0.9 ± 2.4	-0.9 ± 2.4	-0.5 ±2.0
	C	-2.0 ± 3.1	-1.3 ± 3.4	-1.1 ±3.2	-0.7 ± 3.3	0.1 ± 3.0
Hematocrit (%)	P	38 ± 4	32 ± 4	32 ± 4	31 ± 4	30 ± 2
	C	38 ± 4	31 ± 4	31 ± 4	31 ± 4	31 ± 3
PaO_2_ (mmHg)	P	80 ± 14	144 ± 50	168 ± 51	181 ± 41	137 ± 43
	C	93 ± 36	165 ± 52	186 ± 40	192 ± 32	135 ± 33
SvO_2_ (%)*	P	N/A	73 ± 5	72 ± 4	74 ± 5	75 ± 5
	C	N/A	70 ± 6	70 ± 6	71 ± 6	70 ± 6

Values are means ± SD. Interaction between time and group was significant for HR (*p* = 0.012); and insignificant for MBP (*p* = 0.705), MPAP (*p* = 0.979), CI (*p* = 0.402), SVV (*p* = 0.069), lactate (*p* = 0.727), pH (*p* = 0.770), base deficit (*p* = 0.394), hematocrit (*p* = 0.716), PaO2 (*p* = 0.362), and SvO2 (*p* = 0.589).

* *p* < 0.05 between the groups.

1H, 1 h after induction of general anesthesia; 2H, 2 h after induction of general anesthesia; 3H, 3 h after induction of general anesthesia; HR, heart rate; P, prewarming group; C, control group; MBP, mean blood pressure; MPAP, mean pulmonary artery pressure; N/A, not assessed; CI, cardiac index; SVV, stroke volume variation; PaO_2_, arterial partial pressure of oxygen; SvO_2_, mixed venous oxygen saturation.

CI values are from FloTrac™/EV1000™ at the pre-induction period and from the pulmonary artery catheter/Vigilance™ II after induction of anesthesia.

**Table 4:** Postoperative course of patients in the prewarming and control group after off-pump coronary artery bypass surgery.

		Prewarming group	Control group	*p*-value	
		(*n* = 20)	(*n* = 20)		
Amount of chest tube drain (mL/24 h)		654 ± 193	631 ± 162	0.502	
Intraoperative RBC transfusion (unit)		1 (0–2 [0–3])	1 (0–2 [0–4])	0.977	
Postoperative RBC transfusion (unit/24 h)		0 (0–0 [0–2])	0 (0–0 [0–1])	0.689	
Time to extubate after surgery (h)		12 ± 7	15 ± 5	0.079	
Newly developed atrial fibrillation		6 (30%)	7 (35%)	0.736	
Stoke		0 (0%)	2 (10%)	0.487	
Revascularization		2 (10%)	1 (5%)	>0.999	
Wound problem		0 (0%)	0 (0%)		
Postoperative ICU stay (h)		50 ± 38	56 ± 30	0.650	
Postoperative hospital stay (d)		11 ± 4	12 ± 4	0.952	
In-hospital mortality		0 (0%)	1 (5%)	>0.999	
1-year follow-up				
	Number of patients	18 (90%)	18 (90%)	
	Number of total grafts	4 (3–4 [1–5])	4 (3–4 [1–6])	0.655
	Number of patent grafts	4 (3–4 [1–5])	4 (3–4 [1–6])	0.831
	Patency	97% ± 10%	94% ± 20%	0.637

Values are means ± SD, median (IQR [range]) or numbers (%).

RBC, red blood cell; ICU, intensive care unit.

## Discussion

In this study, we demonstrated that prewarming during anesthesia induction effectively preserved T_core_ during the first 3 h after induction. It decreased the incidence of intraoperative hypothermia to less than one-third compared with the control group. However, regarding microcirculatory parameters, neither StO_2_ nor recovery slope was affected by the prewarming. This is the first reported clinical trial to investigate the effect of prewarming on microvascular reactivity measured by StO_2_ with VOT in patients undergoing cardiac surgery.

Inadvertent intraoperative hypothermia develops in about 40% of patients undergoing general anesthesia [21]. If not prevented, up to 70% of patients can become hypothermic after surgery [22]. Intraoperative hypothermia can increase perioperative morbid cardiac events, such as unstable angina, myocardial infarction, or cardiac arrest, in patients undergoing non-cardiac surgery [4]. Hypothermia also impairs platelet function and the coagulation cascade [6, 23], and increases blood loss and transfusion requirements during hip surgery [5]. It has also been related to surgical would infection, and delayed postoperative recovery and hospital stay after non-cardiac surgery [1, 7, 8].

The concept of prewarming in surgical patients started in the early 1990s [24, 25]. Patients undergoing kidney transplantation warmed with forced-air warmer or a circulating-water blanket, with warming starting on induction of anesthesia, were less hypothermic after 3 h of the study period than the control group [24]. Prewarmed patients undergoing hip arthroplasty, using an electric warming blanket before induction of anesthesia, developed less hypothermia than unwarmed patients intraoperatively [25].

The development of intraoperative hypothermia is largely attributable to the redistribution of body heat occurring during the induction of general anesthesia [3, 26]. Thus, to prevent heat loss, body surface warming during this redistribution period may be important. However, in many previous studies on prewarming, active warming was applied before the induction of anesthesia, or commenced on the ward, or in preanesthetic or operating room during the preinduction period [25, 27–31]. In our study, based on our previous study results [32] and to prevent any delay in the surgical procedure, we used a prewarming protocol that included the first 20–30 min after the injection of anesthetics. Thus, we believe that this reduced the incidence of intraoperative hypothermia compared with a previous study [30], in which the prewarming was provided before anesthesia induction for a longer period compared to our study: the incidence of hypothermia was 20% [4/20] vs. 32% [10/31], respectively, and the mean duration of warming time was 35 vs. 72 min. Furthermore, while prewarming provided for up to 50 min during the attachment of monitoring equipment resulted in a mean T_core_ < 36°C after 1 h of induction in another previous study [29], prewarming for 35 min focused on the induction period preserved the mean T_core_ > 36°C during the first 3 h of anesthesia in our study. Moreover, forced air warming started after surgical draping in a previous study did not prevent intraoperative hypothermia during the first hour of anesthesia in patients undergoing non-cardiac surgery [1].

Microcirculation measured using StO_2_ has been advocated to reflect peripheral tissue perfusion [11]. The occlusion and recovery slope, StO_2_-derived parameters following VOT, have been shown to indicate tissue oxygen consumption and microvascular reperfusion adequacy, respectively [10, 33]. Low StO_2_ was associated with poor clinical outcomes in critically ill patients [34], and the reduced recovery slope was related to multiple organ dysfunction and increased mortality in septic patients [13]. In surgical patients, perioperative low StO_2_ was associated with major complications and mortality after non-cardiac surgery [35]. Both the occlusion and recovery slope were higher in survivors than in non-survivors after open cardiac surgery using cardiopulmonary bypass [33]. Similarly, the recovery slope on postoperative day 1 was lower in patients with a composite of major complications than those without after cardiac surgery [17]. In newborn patients treated with therapeutic hypothermia, sluggish microcirculation was observed with a T_core_ of 34°C [18]. Furthermore, during mild hypothermia after cardiopulmonary resuscitation, the recovery slope was initially impaired during the first 24 h, and then recovered to the values of healthy subjects in the next 24 h [19]. Thus, we hypothesized that active warming focused on the period of anesthesia induction would effectively reduce inadvertent intraoperative hypothermia and furthermore enhance the peripheral microcirculation. However, microcirculatory parameters were not affected by the different T_core_ of the two treatment groups in the present study.

Vasoconstriction induced by the use of vasopressors can affect the vascular microcirculation. In previous studies, there were inconsistent data on vasomotor responses caused by the vasoactive agents administered. Administration of noradrenaline did not affect sublingual microperfusion during inhalational general anesthesia [36] or intestinal microcirculation in mice hemorrhagic shock models [37]. StO_2_ measured at the thenar eminence was reduced after noradrenaline infusion but not after phenylephrine administration in normovolemic patients under balanced propofol-remifentanil anesthesia [38]. In the current study, the use of various preoperative and intraoperative cardiovascular drugs was comparable between the groups. Moreover, there was no difference between the groups in the use of vasoactive agents, such as phenylephrine and vasopressin, and the number of patients administered, mean duration, and the maximum dose at each time point for noradrenalin, during the first 3 h after induction of anesthesia as well as throughout the surgery. Thus, the effects of vasopressors on microcirculatory variables, especially on the primary end point–the recovery slope–in our results may be minimal.

Although this was a prospective randomized trial, more diabetic patients were included in the control group than in the prewarming group (12 vs. 6, *p* = 0.057; Table 1). Considering that diabetes mellitus can affect the microvasculature, the skewed distribution of included patients could be a confounding factor, which should be considered when interpreting our data. Moreover, we had more patients who received body surface cooling 3 h after induction in the prewarming group without statistical significance. This trend may have occurred because we strictly controlled body temperature to avoid both hypothermia and hyperthermia in this study, and there was no difference in temperature between the two groups at the end of study. However, it is difficult to draw any conclusion as to whether prewarming may induce hyperthermia because the study was not appropriately powered to address this. It is unclear and beyond of the scope of this study whether intraoperative hyperthermia may cause any clinical effect or whether surface cooling can prevent it during OPCAB surgery. Further studies are needed to investigate these questions.

SvO_2_ has been used as a parameter to describe the adequacy of tissue oxygenation and a low SvO_2_ was associated with poor prognosis in postoperative cardiovascular patients [39–41]. In a previous study, microvascular dysfunction was related to hyperlactatemia and reduced partial pressure of venous oxygen in septic patients [42]. In the current study, there was one mortality case in the control group, but none in the prewarming group. The main cause of death in the deceased case was a cerebral infarction that developed immediately postoperatively. In our results, microcirculatory parameters were similar between the groups; however, SvO_2_ was higher in the prewarming group than the control group, although both groups maintained a mean SvO_2_ ≥ 70% and comparable levels of lactate throughout the surgery. However, the baseline value of SvO_2_ was not available practically in the current study protocol, and the deceased patient in the control group did not show hypothermia at any temperature measurement time point during the study. Thus, it is hard to draw any conclusion from our observation of higher SvO_2_ in the prewarming group, and with respect to whether there is any relationship between prewarming and tissue oxygenation with clinical outcome in the current study.

This study had some limitations. First, the sample size was relatively small. A further study should be conducted to investigate the effects of prewarming on postoperative clinical outcomes and mortality after major cardiovascular surgery. Second, we did not fix the duration of warming time in the treatment group. Thus, our data do not provide results for any particular duration of prewarming. Instead, we used a practical protocol, which can be readily applied to the general practice of anesthesia and preparation for surgery. Prewarming focused on the period of anesthesia induction effectively prevented intraoperative inadvertent hypothermia compared with the control group in our study. Third, we excluded patients with decreased LV contraction. We tried to ensure the uniformity the enrolled population and expected that impaired cardiac function could affect the redistribution of body heat and microcirculation. Thus, the results of our study may not be applied to patients with impaired LV contraction.

In conclusion, prewarming using forced-air warmer during the induction of general anesthesia reduced the incidence of intraoperative hypothermia, but did not improve microvascular reactivity in patients undergoing OPCAB surgery.

## Supporting Information

S1 Fig CONSORT flowchart. (TIF)

S1 File Approval of institutional review board of Seoul National University Hospital. (PDF)

S2 File Study protocol registered at ClinicalTrials.gov. (PDF)

S3 File Original study protocol in original language. (DOC)

S4 File Study protocol in English. (DOCX)

S5 File CONSORT checklist. (DOC)
