# Supplementary figures and images for: Effect of Prewarming during Induction of Anesthesia on Microvascular Reactivity in Patients Undergoing Off-Pump Coronary Artery Bypass Surgery: A Randomized Clinical Trial
Source: PLoS One. 2016 Jul 21;11(7):e0159772. doi: 10.1371/journal.pone.0159772 (PMC4956040; doi:10.1371/journal.pone.0159772)

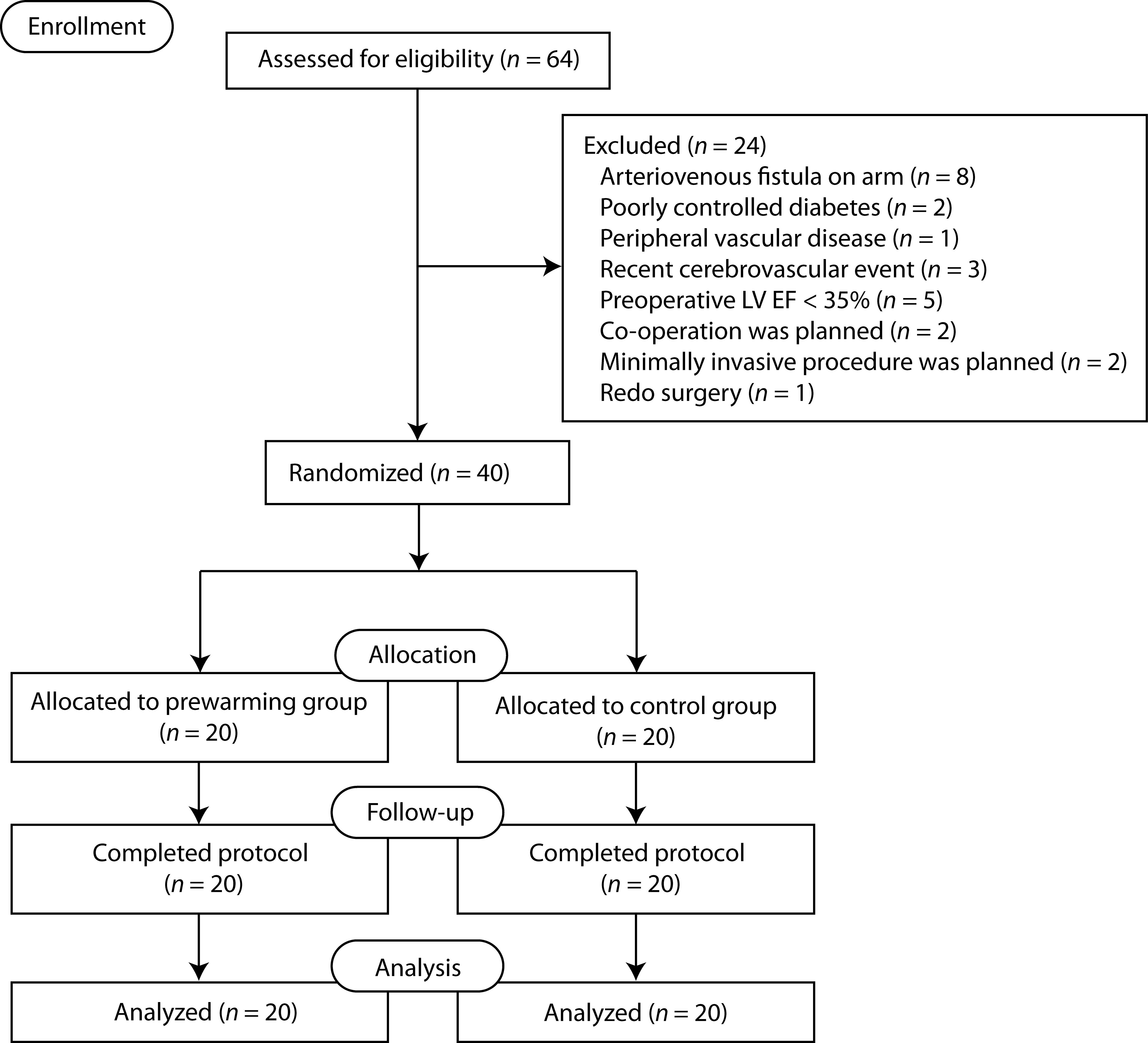

Supplement: S1 Fig — (TIF) [file pone.0159772.s001.tif]
