# Supplementary material for: Effect of Prewarming during Induction of Anesthesia on Microvascular Reactivity in Patients Undergoing Off-Pump Coronary Artery Bypass Surgery: A Randomized Clinical Trial
Source: PLoS One. 2016 Jul 21;11(7):e0159772. doi: 10.1371/journal.pone.0159772 (PMC4956040; doi:10.1371/journal.pone.0159772)
Supplement: S2 File — (PDF) [file pone.0159772.s003.pdf]

ClinicalTrials.gov PRS DRAFT Receipt (Working Version)
Last Update: 01/12/2016 21:07

ClinicalTrials.gov ID: NCT02186210

Study Identification

Unique Protocol ID: Prewarming_OPCAB

Brief Title: Effect of Prewarming on Microcirculatory Response

Official Title: Effect of Prewarming on Microcirculatory Response

Secondary IDs:

Study Status

Record Verification: August 2014

Overall Status: Completed

Study Start: July 2014

Primary Completion: May 2015 [Actual]

Study Completion: May 2015 [Actual]

Sponsor/Collaborators

Sponsor: Seoul National University Hospital

Responsible Party: Sponsor

Collaborators:

Oversight

FDA Regulated?: No

IND/IDE Protocol?: No

Review Board: Approval Status: Approved
Approval Number: H-1306-026-496
Board Name: Seoul National University Hospital Institutional Review Board
Board Affiliation: Seoul National University Hospital
Phone: 82-2-2072-0694
Email: snuhirb@gmail.com

Data Monitoring?: Yes

Plan to Share Data?:

Oversight Authorities: South Korea: Korea Food and Drug Administration (KFDA)

Study Description

Brief Summary: Intraoperative hypothermia may affect tissue microcirculation and can induce myocardial injury, wound infection, and coagulopathy. During off-pump coronary artery bypass surgery without cardiopulmonary bypass or induced hypothermia, maintenance of normothermia is important for clinical outcome. The investigators hypothesized that prewarming during induction of general anesthesia would reduce drop of body temperature and change of peripheral microcirculation.

Detailed Description: Microcirculatory parameters can be obtained from vascular occlusion test. Among those parameters, recovery slope during vascular occlusion test is known to reflect recruitment of microvasculature in response to hypoxic or ischemic insult. In this study, we will compare the recovery slope during vascular occlusion test between prewarming treatment group and control group.

Conditions

Conditions: Coronary Artery Bypass Graft Triple Vessel

Keywords: microcirculation
off-pump coronary artery bypass surgery
tissue oxygen saturation
vascular occlusion test
recovery slope

Study Design

Study Type: Interventional

Primary Purpose: Treatment

Study Phase: N/A

Intervention Model: Parallel Assignment

Number of Arms: 2

Masking: Double Blind (Subject, Caregiver, Investigator)

Allocation: Randomized

Endpoint Classification: Efficacy Study

Enrollment: 40 [Actual]

Arms and Interventions

Arms	Assigned Interventions
Experimental: prewarming<br>prewarming during induction of anesthesia	Device: prewarming<br>recovery slope StO2<br>Other Names:<br>• prewarming by active air heater
No Intervention: control<br>no prewarming during induction of anesthesia	

Outcome Measures

Primary Outcome Measure:

1. recovery slope
   [Time Frame: 3 hours after induction of anesthesia] [Safety Issue: Yes]
   We will compare recovery slope assessed 3 hours after induction of anesthesia to evaluate the effect of prewarming during induction of anesthesia on microcirculation.

Secondary Outcome Measure:

2. tissue oxygen saturation
   [Time Frame: 3 hours after induction of anesthesia] [Safety Issue: No]

Eligibility

Minimum Age: 20 Years

Maximum Age: 85 Years

Gender: Both

Accepts Healthy Volunteers?: No

Criteria: Inclusion Criteria:

- off-pump coronary artery bypass surgery

Exclusion Criteria:

- refuse to enroll
- cannot undergo vascular occlusion test: anatomical abnormality of both arms, severe peripheral vascular disease, presence of A-V fistula
- preoperative left ventricular ejection fraction < 35%
- preoperative continuous infusion of vasopressor or inotropes
- pregnancy

Contacts/Locations

Study Officials: Yunseok Jeon, PhD
Study Principal Investigator
Seoul National University Hospital

Locations: Korea, Republic of
Seoul National University Hospital
Seoul, Korea, Republic of, 110-744

Seoul National University Hospital
Seoul, Korea, Republic of, 110-744

NOTE : Facility 'Seoul National University Hospital' in 'Seoul, Korea, Republic of, 110-744' has been specified multiple times.

References

Citations:

Links:

Study Data/Documents:
