# Supplementary material for: Effect of Prewarming during Induction of Anesthesia on Microvascular Reactivity in Patients Undergoing Off-Pump Coronary Artery Bypass Surgery: A Randomized Clinical Trial
Source: PLoS One. 2016 Jul 21;11(7):e0159772. doi: 10.1371/journal.pone.0159772 (PMC4956040; doi:10.1371/journal.pone.0159772)
Supplement: S3 File — (DOC) [file pone.0159772.s004.doc]

**연구 계획서**

| 수술전 가온이 미세순환 반응성에 미치는 영향  Vascular occlusion test to assess the effect of prewarming on microcirculartory response |
| --- |

Ver 1.3

일시

2013년 IRB 통과시점 – IRB 통과 후 30개월

서울대학교 의과대학 마취통증의학과 교실

부교수 전 윤 석

**연구계획서 요약**

| **연 구 제 목** | 수술전 가온이 미세순환 반응성에 미치는 영향 |
| --- | --- |
| **연 구 목 적** | 수술전 가온이 중심체온 하강을 방지하여 미세순환 반응성을 개선시키는지 알아본다. |
| **연 구 기 관** | 서울대학교병원 마취통증의학과 |
| **연구책임자** | 서울대학교병원 마취통증의학과 전윤석 |
| **연 구 대 상** | 체외순환 없는 관상동맥 우회술을 받는 환자 |
| **연 구 기 간** | IRB 승인 후 30개월 |
| **연 구 방 법** | 수술전 가온을 받는 환자군과 수술전 가온을 받지 않는 환자군으로 무작위로 나눈 후 가온 전후로 근적외선 분광법을 이용한 혈관압박검사를 시행하여 두 군간 미세순환 반응성을 비교한다. 조직산소포화도 recovery slope으로 미세순환 반응성을 비교하도록 한다. |
| **기대효과 및**  **예상결과** | 전신마취 유도 후 재분포 저체온이 발생하고 미세순환의 변화가 일어나는데, 수술전 가온을 통해서 중심체온이 유지되어 미세순환 반응성의 감소도 줄어들 것이다. |

**목 차**

| **1.** |  | 연구 제목 | **--------------------------** | **3** |
| --- | --- | --- | --- | --- |
| **2.** |  | 실시 기관명 및 주소 | **--------------------------** | **3** |
| **3.** |  | 임상 연구자 | **--------------------------** | **3** |
|  | **3.1.** | 연구 책임자 |  |  |
|  | **3.2.** | 공동 연구자 |  |  |
| **4.** |  | 연구 배경 및 목적 | **--------------------------** | **4** |
|  | **4.1.** | 연구 배경 |  |  |
|  | **4.2.** | 연구목적 및 가설 |  |  |
| **5.** |  | 대상질환 및 대상자 | **--------------------------** | **5** |
| **6.** |  | 예상연구기간 | **--------------------------** | **5** |
| **7.** |  | 피험자 선정 및 피험자 수 | **--------------------------** | **5** |
|  | **7.1.** | 피험자의 선정기준, 제외기준 |  |  |
|  | **7.2.** | 피험자 수 |  |  |
| **8.** |  | 임상시험 일정 | **--------------------------** | **6** |
| **9.** |  | 연구 방법 | **--------------------------** | **7** |
|  | **9.1.** | 대상자 선정 |  |  |
|  | **9.2.** | 피험자 동의확보 |  |  |
|  | **9.3.** | 무작위 배정 |  |  |
|  | **9.4.** | 목표 피험자의 수 및 산출 근거 |  |  |
|  | **9.5.** | 임상 시험의 시행계획 |  |  |
|  | **9.6.** | 관찰항목, 검사항목 |  |  |
|  | **9.7.** | 판정 기준 |  |  |
|  | **9.8.** | 안전성 평가 |  |  |
|  | **9.9.** | 통계 분석 원칙 및 방법 |  |  |
| **10.** |  | 피험자 안전보호에 관한 대책 | **--------------------------** | **13** |
| **11.** |  | 연구의 윤리성 확보를 위한 방안 | **--------------------------** | **14** |
| **12.** |  | 모니터링 계획 | **--------------------------** | **14** |
| **13.** |  | 참고문헌 | **--------------------------** | **14** |
| **[별첨1]** | | 피험자 설명문 | **--------------------------** | **19** |
| **[별첨2]** | | 동의서 | **--------------------------** | **21** |
| **[별첨3]** | | 피험자 보상규약 | **--------------------------** | **22** |
| **[별첨4]** | | Case report form | **--------------------------** | **23** |
| **[별첨5]** | | 책임연구자 이력 | **--------------------------** | **25** |
|  | |  |  |  |

**1. 연구 제목**

수술전 가온이 미세순환 반응성에 미치는 영향

**2. 실시 기관명 및 주소**

서울대학교 의과대학 마취통증의학과 교실, 서울특별시 종로구 연건동 28번지

**3. 임상 연구자**

3.1. 연구 책임자

전윤석

부교수

서울특별시 종로구 연건동 28번지, 서울대학교병원 마취통증의학과

Tel. 2072-24674

3.2. 연구담당자

홍덕만

기금조교수

서울특별시 종로구 연건동 28번지, 서울대학교병원 마취통증의학과

Tel. 2072-4911

민정진

진료교수

서울특별시 종로구 연건동 28번지, 서울대학교병원 마취통증의학과

Tel. 2072-3108

김태경

전임의

서울특별시 종로구 연건동 28번지, 서울대학교병원 마취통증의학과

Tel. 2072-3108

조연정

전임의

서울특별시 종로구 연건동 28번지, 서울대학교병원 마취통증의학과

Tel. 2072-3108

**4. 연구 배경 및 목적**

**4.1. 연구 배경**

의도하지 않은 주술기 저체온은 심근허혈, 상처감염, 혈관응고이상 등의 부작용을 일으킬 수 있으며 병원재원기간의 증가와도 연관이 있다. 주술기 저체온이 발생하는 이유는 체열의 재분포, 마취 중 대사 감소, 수술장의 찬 공기 등이 있다.

일반적인 환경에서 피부온도는 중심체온에 비해 2-4도 정도 낮게 유지되는데 이러한 정상적인 중심-말초 체온 경사는 tonic thermoregulatory vasoconstriction에 의해 유지된다. 전신 마취의 영향으로 centrally mediated thermoregulatory vasoconstriction이 억제되고 마취제가 직접적인 말초 혈관 확장을 일으켜 중심체온이 말초로 재분포되면 중심체온은 감소하고 말초체온은 증가한다. 이에 따라 마취 유도 후 수시간에 걸쳐 특징적인 중심체온의 하강이 일어나게 된다.

수술 중에는 피부 가온 접근 부위가 제한적이고 중심체온을 올리기에 시간이 오래 걸리기 때문에 저체온을 막기 어려운 경우가 많은데, 수술전 가온을 통해 중심-말초 체온경사를 줄여 마취유도 후 중심체온의 하강을 감소시킬 수 있다는 보고들이 있다. 또 수술전 가온은 실혈을 감소시키고 상처감염도 줄인다고 보고되었다. 수술중 저체온이 말초순환을 감소시키고 조직의 저산소증을 유발하여 감염에 취약하게 만드는데 수술전 가온이 이를 예방하기 때문으로 생각되고 있다.

미세순환은 조직의 혈류와 국소조직으로 산소전달을 조절하는 중요한 역할을 한다. 미세순환의 변경은 패혈증, 패혈쇼크, 심부전환자, 중환자에서 관찰되었으며 미세순환 변경의 정도는 나쁜 예후와 관련이 있다. 심장수술 중에도 미세순환의 변화가 나타나는데 그 원인은 혈액희석, 저체온, 심폐체외순환, 염증반응, 수술적 자극으로 추정되었다. 수술전 가온은 수술중 저체온을 개선시켜 미세순환에 영향을 줄 것으로 생각되나 이를 증명한 연구는 없다.

체외순환 없는 관상동맥 우회술을 받는 환자에서 수술 중 엄격한 체온관리는 심근 손상을 줄인다고 보고되었고, 체외순환 없는 관상동맥 우회술 중 저체온 방지는 비심장수술에서와 마찬가지로 통상적인 치료목표가 되었다. 본 연구에서는 체외순환 없는 관상동맥 우회술을 받는 환자를 대상으로 수술전 가온을 하여 중심체온과 미세순환의 변화를 감소시킬 수 있는지 알아보고자 한다. 혈관압박검사에서 조직산소포화도 recovery slope은 조직 저산소 자극에 대한 미세현관 recruitment를 반영하는 것으로 생각되고 있다. 본 연구에서 미세순환 반응성은 혈관압박검사 중 조직산소포화도 recovery slope으로 비교하도록 한다.

**4.2. 연구목적 및 가설**

수술전 가온 여부에 따라 미세순환 반응성에 유의한 차이가 있는지 근적외선 분광법을 이용한 혈관압박검사를 통해 알아보고자 한다. 미세순환 반응성은 혈관압박검사 중 조직산소포화도 recovery slope으로 비교하도록 한다. 본 연구의 가설은 ‘수술전 가온을 하는 군과 수술전 가온을 하지 않는 군에서 미세순환 반응성에 차이가 있을 것이다.’ 이다.

**5. 대상질환 및 대상자**

체외순환 없는 관상동맥 우회술을 받는 환자

**6. 예상연구기간**

2013 년 IRB 통과시점 이후 30개월

**7. 피험자 선정 및 피험자 수**

**7.1. 피험자의 선정기준, 제외기준**

**선정기준**

체외순환 없는 관상동맥 우회술을 받는 20세 이상 85세 이하의 환자

**제외기준**

시험에 동의하지 않는 환자

혈관압박검사를 시행할 수 없는 환자 (양측 팔에 해부학적 이상, 중증의 말초혈관폐색성 질환, 화상, 혈액투석관이 있거나 수술을 시행받은 경우)

심각한 말초 혈관 질환 환자

수술 전 심초음파로 측정한 좌심실의 박출계수가 35% 미만인 환자

수술 전 vasopressor 또는 inotropic agent를 지속정주하고 있는 환자

임산부

**중도탈락 및 임상시험 중지 기준**

피험자의 동의 철회

**7.2. 피험자 수**

A군: control 군: 20 명

B군: prewarming을 한 군: 20 명

**8. 임상시험 일정**

2013년 IRB 통과 직후 30개월

2013년 IRB 통과부터 24개월: 임상시험

2013년 IRB 통과부터 30개월: 자료 정리 및 논문 작성

**9. 연구 방법**

**9.1. 대상자 선정**

체외순환 없는 관상동맥 우회술을 받는 환자 중 상기된 선정, 제외기준에 부합되고 피험자가 동의할 경우 대상자로 선정한다.

**9.2. 피험자 동의확보**

별첨된 설명문과 동의서에 따라 연구 담당자가 환자에게 설명한 후 서면 동의를 받는다.

**9.3 무작위 배정**

A군: control 군: 20 명

B군: prewarming을 한 군: 20 명

피험자 등록 전 환자의 진료에 관여하지 않은 의사가 A군과 B군을 각각 2명씩 포함하는 크기가 4인 블록(AABB, BBAA, ABAB, BABA, ABBA, BAAB)이 무작위로 섞인 블록 무작위 배정표를 컴퓨터를 통해 미리 작성한다. 연구와 무관한 제 3자가 무작위배정 운용주체로 참가하여 이 배정표에 따른 순서대로 피험자를 A군이나 B군에 배정하여 시험을 진행한다. 마취담당자는 무작위과정에 관여할 수 없으며 배정표에 접근할 수 없다.

**9.4. 목표 피험자의 수 및 산출 근거**

- 마취유도 3시간 뒤에 혈관압박검사를 통해 측정한 조직산소포화도 recovery slope이 primary end point이다.

- 체외순환없는 관상동맥 우회술을 받은 환자 5명의 pilot study를 행한 결과 마취유도후 3시간 뒤 조직산소포화도 recovery slope의 감소는 평균 1.52 ± 0.65 %/s 였다. 조직산소포화도 recovery slope 감소가 평균값의 50% 이상 차이가 나면 임상적으로 유의하다고 보았다. 제 1종 오류(α)를 0.05, 90%의 power를 가정하면 각 군마다 18 명의 환자가 필요한 것으로 계산되었다.

- 탈락률 10%를 감안하여 군당 20명의 환자를 모집할 예정이다.

**9.5. 임상 시험의 시행계획**

수술전 가온 외에, 모든 마취와 수술방법은 모든 환자에서 동일하게 시행한다. 수술전 가온 여부에 따라 군을 나눈다.

1. A군: control 군: 20 명
2. B군: prewarming을 한 군: 20 명

모든 환자는 수술장에 도착하면 고막체온계를 사용하여 중심체온을 측정하고 피부체온계를 검지 손가락과 forearm에 부착하여 말초체온을 지속적으로 측정한다. InspectraTM StO2 (Hutchinson Technology Inc., MN, USA) probe를 동맥도관이 거치되지 않은 팔의 thenar eminence에 부착하고 공기압지혈대를 동측 팔에 감는다. 팔에 감은 공기압지혈대를 사용하여 혈관압박검사 (vascular occlusion test)를 시행하고 조직산소포화도 recovery slope 등과 같은 지표들을 기록하여, 미세순환을 평가한다.

혈관압박검사 (vascular occlusion test)는 아래와 같은 방법으로 수행하며, InspectraTM StO2 (Hutchinson Technology Inc., MN, USA)를 사용하여 혈관압박검사를 시행한 최근 연구들에서 기술한 방법을 참고하였다.

(1) Thenar eminence에서 측정되는 조직산소포화도가 30초 동안 2%내로 변화하는 안정상태임을 확인한다.

(2) 공기압지혈대가 수축기 혈압보다 50 mmHg를 초과하도록 팽창시키고, 조직산소포화도가 40%로 감소될 때까지 팽창상태를 유지한다.

(3) 공기압지혈대의 공기를 빠르게 0.5초 내로 제거하여, 압박을 중지한다.

(4) 혈관압박검사가 적절히 시행되지 않았다고 판단되는 경우, 5분 후 재측정한다.

무작위배정된 군에 따라서 A군은 통상적인 방식으로 면담요를 덮고, B군은 이불을 덮은 뒤 43도로 설정된 Bair Hugger forced-air heater (Model 505; 3M, St. Paul, MN, USA)를 사용하여 수술전 가온을 시행한다. 피부체온계를 부착한 팔에는 이불을 덮지 않도록 하며 수술전 가온은 폐동맥 카테터 거치가 완료된 뒤 수술을 위한 피부소독을 시작할 때까지 시행한다.

5극 심전도와 맥박산소포화도측정기, 비침습적 혈압감시장치를 부착하고 BIS (bispectral index)와 cerebral oximeter probe를 부착한다. AESCULON® monitor(Electrical velocimetry, Osypka Medical, Berlin, Germany and San Diego, CA, USA) electrode를 경부와 흉곽 좌측에 부착하여 transthoracic impedance를 측정을 통해 유도된 심박출량, 일회박출량, cardiac index를 기록한다. 국소마취 하에 요골동맥에 20G 동맥도관을 거치하여 침습적 혈압측정을 시행하고 FloTrac/EV1000™ system(Edwards Lifesciences, Irvine CA, USA)을 동맥도관에 연결하여 연속적 심박출량, 일회박출량을 측정한다. 내경정맥에 중심정맥관을 거치하고, 7.5 F 폐동맥카테터를 삽입하고 지속적 심박출량 측정장치(Vigilance 2 monitor, Edwards Lifesciences, CA, USA)에 연결한다. 정맥혈가스분석(venous blood gas analysis, VBGA)을 시행하여 혼합정맥혈 산소포화도(mixed venous oxygen saturation, SvO2)의 생체 내 보정(in vivo calibration)을 시행하고 SvO2를 연속적으로 감시한다. 수술 중 평균동맥압, 심박수, 중심정맥압, 평균폐동맥압, 심박출량, SvO2는 연속적으로 표시된다.

마취유도 중과 수술 중 발생하는 저혈압에 대해서, phenylephrine, ephedrine, vasopressin, calcium chloride 등을 정맥주사하거나 dobutamine, norepinephrine, nitroglycerin을 지속 정주한다. Hematotcrit은 26-30%로 유지되도록 수혈한다. 마취의 유지를 위해, propofol 효과처농도가 1-4 ㎍/ml가 되도록, remifentanil의 효과처농도가 5-12 ng/ml가 되도록, orchestra base intensive (Fresenius Kabi, Stans, Switzerland)를 이용하여 목표농도지속정주법을 시행한다. 근이완을 위해 vecuronium 0.04-0.08 mg/kg/h로 지속정주한다.

혈관압박검사는 마취유도 1시간 후, 2시간 후, 3시간 후, 수술 종료 시점에 다시 시행하여 조직산소포화도 recovery slope 등을 기록하여 미세순환을 평가한다.

**9.6. 관찰항목, 검사항목**

- 기본항목

1. 성별, 나이, 체중, 신장, Body mass index, 수술명, 진단명, 기저질환 (당뇨, 고혈압, 폐질환, 뇌혈관질환, 부정맥, 경동맥 협착 또는 폐쇄질환, 이상지질혈증), 흡연력 (current smoker, former smoker, never smoker), Euro score, STS score, NYHA class
2. 동맥도관의 위치: 좌측 또는 우측
3. 수술 전 지속정주하고 있는 약제명과 용량
4. 수술 전 복용 중인 약물 (beta-blocker, calcium channel blocker, diuretics, angiotensin-converting enzyme inhibitor, angiotensin 2-receptor blocker, insulin 등)
5. 수술 전 시행한 심초음파 상 EF(%)

- 혈역학적 지표 및 환기 지표

1. 수축기압, 이완기압, 평균압, 심박수, 동맥혈산소포화도
2. 수축기동맥압, 이완기동맥압, 평균동맥압, 수축기폐동맥압, 이완기폐동맥압, 평균폐동맥압, 중심정맥압, 심박출량지수(AESCULON®, FloTrac/EV1000™, Vigilance 2 monitor), SvO_2_
3. 체온(중심체온, 피부체온)
4. Peak inspiratory pressure, plateau pressure, mean airway pressure, tidal volume, respiratory rate, FIO_2_, PaO_2_/FIO_2_
5. ABGA: Hct, pH, pCO_2_, pO_2_, HCO_3_^-^, Base excess, SaO_2_, glucose, lactate
6. Bispectral index score, cerebral oximeter score
7. Transthoracic echocardiography 및 transesophageal echocardiography로 측정한 혈역학적지표: cardiac output, stroke volume

- 혈액검사

ROTEM® (Pentapharm GmbH, Munchen, Germany)

- InspectraTM StO_2_ (Model 650; Hutchinson Technology Inc., MN, USA) 장치를 통해 얻은 지표: 2초마다 데이터가 자동으로 기록된다.

1. StO_2_ (%)
2. THI (tissue hemoglobin index)
3. 혈관압박검사를 시행하는 동안 StO_2_ (%)의 변화곡선을 기록한 후, Recovery slope (%/s) 을 계산한다. 혈관압박검사를 시행한 과거논문을 참조하여 계산한다.

- 혈역학적 지표 및 환기 지표, 혈액검사, InspectraTM StO_2_ 데이터 수집 시점
  - 가온 전, 가온 종료, 1시간 뒤, 2시간 뒤, 3시간 뒤, 수술 종료 시

**9.7. 판정 기준**

마취유도 3시간 뒤 혈관압박검사를 통해 측정한 조직산소포화도 recovery slope이 primary end point로 두 군간 Independent t-test 또는 Wilcoxon-Mann-Whitney test 에서 P 값이 <0.05인 경우 통계학적으로 유의한 것으로 판단한다.

**9.8. 안전성 평가**

본 시험에 참여한 모든 환자를 대상으로 안전성 평가를 실시한다.

연구 시행 중에 이상반응이 발생했을 경우, 연구에 사용된 시험 방법과의 인과 관계 유무와 모든 이상 반응을 기록하고 추후 중증도, 중대성, 기간, 그리고 시험 방법과의 인과관계를 평가한다. 이상반응에 대한 처치 및 결과 역시 기록한다. 이상 반응은 시험 기간 중의 계획된 검진과 검사의 소견 이외에도 비정상적인 검진이나 필요에 따른 추가적인 검사와 검진에 의해 평가하고 즉각적인 조치를 시행한다. 임상병리검사 자료에 대해서는 변수의 특성에 따라 치료 전, 후의 군내 비교 등 적절한 통계적 방법을 이용하여 분석하고, 이상반응의 빈도, 발현율, 각각의 목록, 심각한 정도 및 시험 방법과의 인과관계 등을 제시하며, 필요한 경우 그래프 형태로 보고한다.

1)중증도

이상반응은 아래의 정의에 따라 경증, 중등증, 중증으로 구분된다.

경증은 일반적이고 일시적이고 일상적인 활동을 방해하지 않는다.

중등증은 약간의 불편함을 초래하거나 일상적인 활동을 방해한다.

중증은 일상적인 활동을 수행할 수 없다.

2)인과 관계

인과 관계는 ‘관련 없을 것으로 생각됨’, ‘관련 있을 가능성 있음’, ’가능성 많음’, ’명백히 관련 있음’, 또는 ‘관련성을 확인하기 어려움’으로 구분된다.

3)이상 반응의 분류

Forced air warmer를 이용한 가온 방식은 주술기 환자 체온 유지에 널리 사용되고 있는 방식으로 수술전 가온이 오래 지속될 경우 환자가 땀이 나거나 열불편감을 느낄 수 있으나 본 연구에서 계획하고 있는 38도 30분간의 가온이 환자에게 추가적인 합병증을 발생시킬 가능성은 거의 없다. 상지에 공기압지혈대와 같은 압박띠를 감아서 허혈을 유발하는 방법은 무혈적 수술 시야 확보를 위해 상지 수술시 널리 사용되고 있는 방법이다. 1-3시간 이내의 허혈은 합병증 없이 안전하게 사용할 수 있다고 알려져 있다. 드물게 정맥염, 점상출혈, 부종, 말초신경염들을 유발할 수 있으나 그 빈도는 매우 낮다. 따라서 본 연구에서 계획하고 있는 혈관압박검사는 3-4 분 동안의 상지의 허혈을 유발하는 것으로 부작용이 거의 없는 방법이며, 환자에게 추가적인 합병증을 발생시킬 위험은 거의 없다고 할 수 있다. AESCULON® monitor(Electrical velocimetry)는 electrode를 경부와 흉곽 좌측에 부착하여 transthoracic impedance를 측정하여 심박출량을 계산하는 장치로 환자에게 추가적인 위해를 줄 가능성은 거의 없다.

**9.9. 통계 분석 원칙 및 방법**

통계 프로그램은 SPSS 21.0을 사용한다. 각 항목에 대해 A 군과 B 군 간 비교 분석을 아래와 같은 방법으로 시행한다. Per protocol 군에 따라서 분석한다. 결측치가 있거나 선정기준 및 제외기준에 맞지 않는 환자가 참여하였음을 나중에 발견하게 되는 경우 통계분석에서 제외하며, 임상시험 결과 보고서에 피험자들이 왜 제외되었는지 그 이유를 쓰고 요약하도록 한다. 두 군간 성별, 동맥도관의 위치, 기저질환의 유무, 흡연력의 비교는 Chi-square test 또는 Fisher’s exact test를 이용하여 분석한다. 나이, 체중, 신장, Body mass index, InspectraTM StO2 (Hutchinson Technology Inc., MN, USA)를 통해 간헐적으로 기록한 지표 또는 혈역학적 지표, 심박출량 및 일회박출량, 환기지표 등은 연속변수로 두 군간 Independent t-test 또는 Wilcoxon-Mann-Whitney test를 이용하여 특정시점의 값을 분석하거나, RMANOVA 또는 Generalized estimating equation (GEE)를 사용하여 여러 시점을 동시에 분석한다. P 값이 <0.05인 경우 통계학적으로 유의한 것으로 판단한다.

**10. 피험자 안전보호에 관한 대책**

Forced air warmer를 이용한 가온 방식은 주술기 환자 체온 유지에 널리 사용되고 있는 방식으로 수술전가온이 오래 지속될 경우 환자가 땀이 나거나 열불편감을 느낄 수 있으나 본 연구에서 계획하고 있는 38도 30분간의 가온이 환자에게 추가적인 합병증을 발생시킬 가능성은 거의 없다. 미세순환 반응성을 측정하기 위한 상지 압박 역시 그 시간이 5분 이내로 짧아서 추가적 합병증을 유발할 가능성이 거의 없다. 상지에 공기압지혈대와 같은 압박띠를 감아서 허혈을 유발하는 방법은 무혈적 수술 시야 확보를 위해 상지 수술시 이미 널리 사용되고 있는 방법이다. 1-3 시간 이내의 허혈은 합병증 없이 안전하게 사용할 수 있다고 알려져 있다. 드물게 정맥염, 점상출혈, 부종, 말초신경염들을 유발할 수 있으나 그 빈도는 매우 낮다. 또한 마취 유도 및 유지 과정은 숙련된 마취과 전문의가 함께 감시하므로 합병증이 발생하더라도 적절한 조치를 취하게 되므로 환자에게 추가 위험의 가능성은 매우 적을 것이라 생각한다. AESCULON® monitor(Electrical velocimetry)는 electrode를 경부와 흉곽 좌측에 부착하여 transthoracic impedance를 측정하여 심박출량을 계산하는 장치로 환자에게 추가적인 위해를 줄 가능성은 거의 없다.

피험자의 의무기록을 포함한 사적정보의 보호를 위해 다음의 내용을 실천한다.

1) 데이터는 미리 준비한 case report form에 기록하며, 즉시 잠금장치가 있는 연구실에 비밀번호가 걸린 파일로 연구 종료 후 1년 동안 보관한다.

2) 연구파일에 접근할 수 있는 사람은 권한을 가진 일부 연구원으로 제한한다.

3) 수집되는 자료의 불필요한 개인식별자는 제거한다. 특히, 증례기록서에는 환자의 이름, 주민등록번호, Chart No. 등을 기재하지 않도록 하며, 신상정보와 연결된 식별자 코드는 별도로 관리한다.

**11. 연구의 윤리성 확보를 위한 방안**

2008 헬싱키 선언에 입각하여, 피험자 또는 보호자에게 연구의 목적과 연구 참여 중 일어날 수 있는 정신적, 신체적 위해를 충분히 설명한 후 피험자 (또는 보호자) 로부터 서면동의서를 받을 예정이다. 피험자, 피험자의 담당의사, 시험 참여자 이외에는 피험자의 시험 참여 여부나 치료 경과에 대해 알지 못하게 하며, 피험자의 신원을 파악할 수 있는 기록은 비밀로 보장될 것이다. 수집되는 자료의 불필요한 개인식별자는 제거하고, 특히, 증례 기록서에는 환자의 이름, 주민등록번호, 차트 번호 등을 기재하지 않도록 하며, 신상정보와연결된 식별자 코드는 별도로 관리할 것이다. 환자와 관련된 사진을 제출할 때는 환자의 신원을 알 수 없도록 할 것이며 조금이라도 신원이 노출될 가능성이 있는 경우에는 이에 대한 서면 동의를 받았음을 명시할 것이다. 피험자의 검진 기록은 비밀이 유지되고 다른 곳으로 이동되지 않을 것이며, 본 연구의 진행 여부를 감독 받기 위해 감독 기관으로 보내어질 수 있다. 본 연구는 병원윤리위원회의 윤리규정을 준수할 것이다.

**12. 모니터링 계획**

본 연구는 피험자의 권리와 복지를 보호하기 위해서 모든 피험자의 증례기록서와 근거문서를 작성 보관하며, 추후 본 연구와 관계가 없는 마취통증의학과 서정화 교수가 임상시험 계획서와 GCP 준수 여부를 확인하고, 증례기록서에 기입된 정보가 정확함을 확인하기 위해 근거자료를 대조하여 감독할 수 있도록 하며, 이로써 자료의 정확성과 완전성 그리고 검증가능성을 뒷받침 하도록 한다.

**13. 참고문헌**

**1 Winkler M, Akça O, Birkenberg B, et al. Aggressive warming reduces blood loss during hip arthroplasty. *Anesthesia & Analgesia* 2000; 91: 978-84**

**2 Sessler DI. Complications and treatment of mild hypothermia. *Anesthesiology* 2001; 95: 531-43**

**3 Andrzejowski J, Hoyle J, Eapen G, Turnbull D. Effect of prewarming on post-induction core temperature and the incidence of inadvertent perioperative hypothermia in patients undergoing general anaesthesia. *British journal of anaesthesia* 2008; 101: 627-31**

**4 Kim JY, Shinn H, Oh YJ, Hong YW, Kwak HJ, Kwak YL. The effect of skin surface warming during anesthesia preparation on preventing redistribution hypothermia in the early operative period of off-pump coronary artery bypass surgery. *European journal of cardio-thoracic surgery* 2006; 29: 343-7**

**5 Melling AC, Ali B, Scott EM, Leaper DJ. Effects of preoperative warming on the incidence of wound infection after clean surgery: a randomised controlled trial. *The Lancet* 2001; 358: 876-80**

**6 Lenhardt R, Marker E, Goll V, et al. Mild intraoperative hypothermia prolongs postanesthetic recovery. *Anesthesiology* 1997; 87: 1318-23**

**7 Sessler DI, Todd MM. Perioperative heat balance. *Anesthesiology* 2000; 92: 578**

**8 Frank SM, Fleisher LA, Breslow MJ, et al. Perioperative maintenance of normothermia reduces the incidence of morbid cardiac events. *JAMA: the journal of the American Medical Association* 1997; 277: 1127-34**

**9 Just B, Trévien V, Delva E, Lienhart A. Prevention of intraoperative hypothermia by preoperative skin-surface warming. *Anesthesiology* 1993; 79: 214-8**

**10 Nesher N, Zisman E, Wolf T, et al. Strict thermoregulation attenuates myocardial injury during coronary artery bypass graft surgery as reflected by reduced levels of cardiac-specific troponin I. *Anesthesia & Analgesia* 2003; 96: 328-35**

**11 Kurz A, Sessler DI, Lenhardt R. Study of wound infections and temperature group: Perioperative normothermia to reduce the incidence of surgical-wound infection and shorten hospitalization. *N Engl J Med* 1996; 334: 1209-15**

**12 Atasever B, Boer C, Goedhart P, et al. Distinct alterations in sublingual microcirculatory blood flow and hemoglobin oxygenation in on-pump and off-pump coronary artery bypass graft surgery. *Journal of cardiothoracic and vascular anesthesia* 2011; 25: 784-90**

**13 Trzeciak S, McCoy JV, Dellinger RP, et al. Early increases in microcirculatory perfusion during protocol-directed resuscitation are associated with reduced multi-organ failure at 24 h in patients with sepsis. *Intensive care medicine* 2008; 34: 2210-7**

**14 Spanos A, Jhanji S, Vivian-Smith A, Harris T, Pearse RM. Early microvascular changes in sepsis and severe sepsis. *Shock* 2010; 33: 387-91**

**15 den Uil CA, Lagrand WK, Spronk PE, et al. Impaired sublingual microvascular perfusion during surgery with cardiopulmonary bypass: a pilot study. *The Journal of thoracic and cardiovascular surgery* 2008; 136: 129**

**16 Daniel De Backer M, Dubois M-J, Schmartz D, et al. Microcirculatory alterations in cardiac surgery: effects of cardiopulmonary bypass and anesthesia. *Ann Thorac Surg* 2009; 88: 1396-403**

**17 De Backer D, Creteur J, Dubois M-J, Sakr Y, Vincent J-L. Microvascular alterations in patients with acute severe heart failure and cardiogenic shock. *American heart journal* 2004; 147: 91-9**

**18 Bauer A, Kofler S, Thiel M, Eifert S, Christ F. Monitoring of the sublingual microcirculation in cardiac surgery using orthogonal polarization spectral imaging: preliminary results. *Anesthesiology* 2007; 107: 939-45**

**19 De Backer D, Ospina-Tascon G, Salgado D, Favory R, Creteur J, Vincent J-L. Monitoring the microcirculation in the critically ill patient: current methods and future approaches. *Intensive care medicine* 2010; 36: 1813-25**

**20 Sakr Y, Dubois M-J, De Backer D, Creteur J, Vincent J-L. Persistent microcirculatory alterations are associated with organ failure and death in patients with septic shock*. *Critical care medicine* 2004; 32: 1825-31**

**21 Sanders J, Toor IS, Yurik TM, Keogh BE, Mythen M, Montgomery HE. Tissue oxygen saturation and outcome after cardiac surgery. *American Journal of Critical Care* 2011; 20: 138-45**

**22 De Blasi RA, Palmisani S, Alampi D, et al. Microvascular dysfunction and skeletal muscle oxygenation assessed by phase-modulation near-infrared spectroscopy in patients with septic shock. *Intensive care medicine* 2005; 31: 1661-8**

**23 Creteur J, Carollo T, Soldati G, Buchele G, De Backer D, Vincent J-L. The prognostic value of muscle StO2 in septic patients. *Intensive care medicine* 2007; 33: 1549-56**

**24 Georger J-F, Hamzaoui O, Chaari A, Maizel J, Richard C, Teboul J-L. Restoring arterial pressure with norepinephrine improves muscle tissue oxygenation assessed by near-infrared spectroscopy in severely hypotensive septic patients. *Intensive care medicine* 2010; 36: 1882-9**

**25 Gómez H, Torres A, Polanco P, et al. Use of non-invasive NIRS during a vascular occlusion test to assess dynamic tissue O2 saturation response. *Intensive care medicine* 2008; 34: 1600-7**

**[별첨1]**

**대상자 설명문**

**본 임상 시험의 목적**

근적외선 분광법을 이용한 혈관압박검사를 통해 수술전 가온장치를 통한 체온 관리 여부에 따라서 미세순환 반응성에 유의한 차이가 있는지 알아보는 연구입니다. 과거 연구에 의하면 수술 중 발생하는 미세순환 변경의 정도가 클수록 예후가 나쁜 것으로 알려져 있으므로 미세순환 변경의 정도를 감소시킨다면 수술 후 합병증의 발생을 예방할 수 있을 것으로 추측됩니다.

**본 임상 시험의 대상**

체외순환 없는 관상동맥 우회술을 받는 20세 이상 80 세 이하의 대상자에게 시험에 참여를 요청합니다. 총 40명이 본 연구에 참여할 예정이며 동일한 비율로 각 군에 배정받게 됩니다.

**자발적인 참여**

연구의 참여는 본인의 자발적인 의사에 의해서만 가능합니다. 만일 참여를 원하지 않으신다고 하여도 추후의 치료과정에는 어떤 영향도 미치지 않을 것입니다.

**시험은 어떻게 진행되나요?**

본 연구에서는 수술전 가온 여부에 따라 대상자를 A군 또는 B군으로 무작위 배정합니다. A군에서는 통상적인 수술전 체온 관리를 시행하고 B군에서는 30분 가량 가온장치를 통해 체온관리를 합니다. 두 가지 방법 모두 기존에 사용하고 있는 방법입니다. 이외의 마취 및 수술 과정은 모든 대상자들께 동일하게 적용되며, 심장수술을 위해, 통상 행해지고 있는 전신마취의 유도 및 유지가 시행됩니다. 전신마취 중, 대상자의 일측 손의 무지구 (thenar eminence)에 비침습적 장치인 InspectraTM StO2 (Model 650; Hutchinson Technology Inc., MN, USA)를 연결하여 조직의 산소포화도를 비롯한 미세순환을 평가하는 지표들을 기록합니다.

**가능한 부작용은 무엇이 있나요?**

수술전가온을 하는 동안 땀이 나거나 열불편감을 느낄 수 있으나 본 연구에서 계획하고 있는 38도 30분의 가온이 대상자에게 추가적인 합병증을 발생시킬 가능성은 거의 없습니다. 조직의 미세순환을 평가하기 위해 혈관압박검사를 시행하는데, 이는 3-4 분 동안의 상지의 허혈을 유발하는 것으로 부작용이 거의 없는 방법입니다. 또한 상지에 공기압지혈대와 같은 압박띠를 감아서 허혈을 유발하는 방법은 무혈적 수술 시야 확보를 위해 상지 수술시 널리 사용되고 있는 것으로, 1-3 시간 이내의 허혈은 합병증 없이 안전하게 사용할 수 있다고 알려져 있습니다. 드물게 정맥염, 점상출혈, 부종, 말초신경염들을 유발할 수 있으나 그 빈도는 매우 낮습니다. 본 연구에서 계획하고 있는 혈관압박검사는 매우 짧은 기간동안 행해지는 것으로, 대상자에게 추가적인 합병증을 발생시킬 위험은 거의 없다고 할 수 있습니다. 임피던스를 이용한 비침습적 심박출량 측정장치 전극을 통해 임피던스를 측정하여 심박출량을 계산하는 장치로 환자에게 위해를 줄 가능성은 거의 없습니다. 또한 마취 유도 및 유지 과정은 숙련된 마취과 전문의가 함께 감시하므로 합병증이 발생하더라도 적절한 조치를 취하게 되므로 대상자에게 추가 위험이 생길 여지는 극히 적습니다.

**시험참여에 따른 부담과 그 보상**

본 연구에 쓰이는 연구방법은 선행된 연구에서 안전하게 사용되어 왔고, 부작용이 보고되지 않은 방법이므로 부작용이 나타날 위험은 거의 없다고 볼 수 있습니다. 시험 참여에 따르는 추가적인 보상이나 금전적 혜택은 제공되지 않습니다. 대상자께서 원하신다면 시험 도중 언제라도 중도에 참여를 포기할 수 있으며, 추후 치료과정에는 어떠한 영향도 없을 것입니다. 임피던스를 이용한 비침습적 심박출량 측정장치로 인하여 대상자에게 추가적인 금전적인 부담은 없습니다.

**피험자와 다른 사람들에 대한 이익**

앞서 설명하였듯이 본 연구참여로 인해 금전적으로나 다른 어떠한 형태로도 대상자께서 받는 이익이나 손해는 없습니다. 본 연구의 결과가 나왔을 때, 이를 학문적으로 학계에 보고하여 결과를 향상시키는데 도움을 줄 수 있다면, 이로 인해 수많은 환자들에게 긍정적인 영향을 줄 수 있을 것으로 생각됩니다.

**비밀 보장**

본 연구 결과는 학술 목적으로만 이용되며 학술적인 보고를 위하여 외부로 발표될 수 있습니다. 대상자와 대상자의 담당의사, 그리고 시험 참여자 이외에는 대상자의 시험 참여 여부나 치료 경과에 대해서는 알지 못할 것입니다. 또한 피험자의 신원을 파악할 수 있는 기록은 비밀로 보장될 것이며, 임상시험의 결과가 출판될 경우 피험자의 신원은 비밀상태로 유지될 것입니다. 대상자의 검진 기록은 비밀이 유지되고 다른 곳으로 이동되지 않을 것이며, 본 연구의 진행 여부를 감독 받기 위해 감독 기관으로 보내어 질 수 있습니다.

대상자께서 동의하신다면 대상자의 진료를 위해 다른 의사에게 시험의 참여 사실이 통보될 것입니다. 귀하의 기록은 연구 종료 후 1년 동안 보관될 것이며 추후 모든 자료는 폐기될 것입니다.

임상시험 참여와 관련하여 귀하의 권익에 관한 추가적인 정보를 얻고자 하는 경우, 서울대학교병원 의학연구윤리심의위원회(02-2072-0694)로 문의하여 주시기 바랍니다. 제공되는 설명문 및 동의서 사본을 참고하십시오.

**담당 의사 이름과 연락처:**

연구책임자: 서울대학교병원 마취통증의학과 부교수 전윤석 02-2072-2467

연구담당자: 서울대학교병원 마취통증의학과 기금조교수 홍덕만 02-2072-4911

서울대학교병원 마취통증의학과 진료교수 민정진 02-2072-3108
서울대학교병원 마취통증의학과 전임의 김태경 02-2072-3108

**[별첨2]**

**동 의 서**

수술전 가온이 미세순환 반응성에 미치는 영향

1. 본인은 설명문을 읽음과 동시에 담당 의사로부터 구두로 자세히 설명을 듣고 의문 사항에 대해서 충분히 의논하였습니다.
2. 본인은 발생 가능한 위험에 관하여 들었으며 나의 질문에 만족할 만한 답변을 얻었습니다.
3. 본인은 언제든지 중도 탈락을 결정할 수 있고, 이러한 결정이 향후 진료에 있어 불이익을 받지 않을 것을 알고 있습니다.
4. 본인은 이 동의서에 서명함으로써 현행 법률과 규정이 허용하는 범위 내에서 연구와 관련된 정보가 수집된다는 것을 알고 있습니다.
5. 본인은 이 동의서 사본을 받을 것을 알고 있습니다.

================================================================

본인(대상자가 동의 의사를 표명하기 어려운 상황에 있을 때는 대상자에 대해 책임질 수 있는 위치에 있는 법적 대리인)은 담당 의사로부터 사전 설명을 들은 후 이에 자발적으로 동의합니다.

| 날짜 ( 년/ 월/ 일) |  | |  | | 대상자 성명____________ | 서명________ |
| --- | --- | --- | --- | --- | --- | --- |
| 날짜 ( 년/ 월/ 일) | |  | | 대리인인 경우  대상자와의 관계_________ | 대리인 성명____________ | 서명________ |
| 날짜 ( 년/ 월/ 일) | |  | |  | 설명한 의사 성명_________ | 서명________ |

**[별첨3]**

**피험자 보상 규약**

1. 본 임상시험의 연구자는 임상시험기간 동안 합의된 임상시험계획서에 따라 시행된 과정의 결과로 피험자에게 피해가 발생하였을 경우 책임을 진다. 합병증이나 부작용이 발생할 경우 합법적으로 요구되는 치료를 소홀히 하여 발생한 피해에 대해서 책임을 진다. 단, 연구자가 책임을 지는 경우는 피험자에게 발생하는 피해와 임상연구 사이에 인과관계가 없음을 입증하지 못하는 경우로 제한된다.

2. 다음의 경우에는 보상 대상에서 제외된다.

- 연구자의 책임하에 시행되지 않은 과정 중 발생한 부작용에 의한 손상

- 연구와는 무관하게 피험자가 원래부터 가지고 있었던 기저질환의 악화

- 피험자의 부주의에서 초래된 손상

- 임상 실험 도중 대상에서 제외되어 연구가 중단된 이후 발생한 손상

3. 임상시험 책임자는 피험자가 본 임상시험에 의해 어떠한 불이익이라도 받지 않도록 관계법규와 규범, 상호 합의한 임상시험계획서의 내용을 충실히 준수하는 등 최선을 다해야 한다. 그러나 이러한 노력에도 불구하고 연구자가 시행한 임상 시험에 의해 피험자가 피해(상해 또는 부작용 발생)를 입게 된 경우는 아래의 보상평가기준에 따라 합리적인 치료비 또는 보상금을 지급한다.

[보상평가 기준]

치료비 또는 보상금은 피해의 본질, 정도, 기간, 지속성여부 등에 따라 이를 치료 또는 보상할 수 있는 적절한 액수여야 하며 한국법정에서 유사피해에 대해 일반적으로 지급되도록 하는 것과 동일한 수준으로 한다. 보상수준에 대해 피험자와 연구자 사이에 이견이 있을 경우에는 우선적으로 양 당사자의 합의 하에 선정한 전문가로부터 자문을 구하도록 하고, 자문을 받을 경우에는 자문내용에 따르기로 한다.

본 임상시험에서 피험자가 불이익을 받지 않도록 주의를 기울이고, 피험자가 입는 피해에 대하여 상기 내용에 의거하여 책임질 것을 서약합니다.

2013. 05. 연구 책임자

서울대학교병원 마취통증의학과

부교수 전윤석


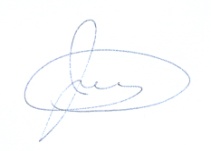


**[별첨]**

**Case Report Form -** 수술전 가온이 미세순환 반응성에 미치는 영향

날짜 ____________ 일련번호 _____________

□ A군: Control

□ B군: 수술전가온 (시작시간 ____________ 종료시간 ____________ 총가온시간 ____________ )

| 기본항목 | |
| --- | --- |
| 성별 |  |
| 나이 |  |
| 체중 (kg) |  |
| 신장 (cm) |  |
| Body mass index |  |
| 수술명 |  |
| 진단명 |  |
| 기저질환(당뇨병, 고혈압, 폐질환, 뇌혈관질환, 부정맥, 경동맥협착 및 폐쇄질환, 이상지질혈증 등) |  |
| 흡연력 | Current / former / never |
| Euro SCORE |  |
| STS SCORE |  |
| NYHA class |  |
| 수술 전 시행한 심초음파 상 EF (%) |  |
| 수술 전 복용중인 약물 |  |
| Beta-blocker |  |
| Calcium channel blocker |  |
| Diuretics |  |
| Angiotensin-converting enzyme inhibitor |  |
| Angiotensin 2-receptor blocker |  |
| Insulin |  |
| 수술 전 지속정주하고 있는 약제 |  |
| 종류 |  |
| 정주속도 |  |
| 동맥도관의 위치 | Lt. / Rt. |

| 이상 반응 평가 | | | | |
| --- | --- | --- | --- | --- |
| 이상 반응 | 중등도 | 기간 | 처치 | 인과관계 |
|  |  |  |  |  |
|  |  |  |  |  |

Date Principle Investigator ____________________

|  | **가온 전** | **가온 종료** | **1시간 뒤** | **2시간 뒤** | **3시간 뒤** | **수술 종료** |
| --- | --- | --- | --- | --- | --- | --- |
| **SBP** |  |  |  |  |  |  |
| **DBP** |  |  |  |  |  |  |
| **MBP** |  |  |  |  |  |  |
| **HR** |  |  |  |  |  |  |
| **SpO_2_** |  |  |  |  |  |  |
| **SAP** |  |  |  |  |  |  |
| **DAP** |  |  |  |  |  |  |
| **MAP** |  |  |  |  |  |  |
| **sPAP** |  |  |  |  |  |  |
| **dPAP** |  |  |  |  |  |  |
| **mPAP** |  |  |  |  |  |  |
| **CVP** |  |  |  |  |  |  |
| **CI (AESCULON®)** |  |  |  |  |  |  |
| **CI (FloTrac/EV1000™)** |  |  |  |  |  |  |
| **CI (Vigilance 2)** |  |  |  |  |  |  |
| **SV** |  |  |  |  |  |  |
| **ScvO_2_** |  |  |  |  |  |  |
| **Temp** |  |  |  |  |  |  |
| **BIS** |  |  |  |  |  |  |
| **Cerebral oximeter (Lt/Rt)** |  |  |  |  |  |  |
| **PIP** |  |  |  |  |  |  |
| **Plateau pressure** |  |  |  |  |  |  |
| **Mean airway pressure** |  |  |  |  |  |  |
| **Tidal volume** |  |  |  |  |  |  |
| **Respiratory rate** |  |  |  |  |  |  |
| **FIO_2_** |  |  |  |  |  |  |
| **PaO2/FIO2** |  |  |  |  |  |  |
| **Hct** |  |  |  |  |  |  |
| **pH** |  |  |  |  |  |  |
| **pCO_2_** |  |  |  |  |  |  |
| **pO_2_** |  |  |  |  |  |  |
| **HCO_3_^-^,** |  |  |  |  |  |  |
| **Base excess** |  |  |  |  |  |  |
| **SaO_2_** |  |  |  |  |  |  |
| **glucose** |  |  |  |  |  |  |
| **lactate** |  |  |  |  |  |  |
| **StO_2_** |  |  |  |  |  |  |
| **THI** |  |  |  |  |  |  |
| **Recovery slope** |  |  |  |  |  |  |

**[별첨5]**

**책임 연구자 최근 이력 및 기타 경력**

**연구 책임자의 최근 이력 또는 기타 경력에 관한 문서**

1.개인 사항

성명: 전윤석 병역: 예비역

주민등록 번호: 690721-1041911

주소: 서울 특별시 송파구 방이동89 올림픽선수촌아파트 303-1904

2.학력

| 학위과정명 | 기 간 | | 학부(과) | 전공 | 학위명 | 대학(원)  수여기관 |
| --- | --- | --- | --- | --- | --- | --- |
|  | 입학연월일 | 졸업연월일 |  |  |  |  |
| 학 사 | 1988.3 | 1994.2 | 의학과 | 의학과 | 의학사 | 서울대학교 |
| 석 사 | 1998.3 | 2003.2 | 의학과 | 마취과학 | 의학석사 | 서울대학교 |
| 박 사 | 2003.3 | 2005.2 | 의학과 | 마취과학 | 의학박사 | 서울대학교 |

※ 의사면허번호 : 53042 (취득일 1994.3), 전문의자격번호: 2013 (취득일 1999.3)

3.경력

| 기 간(년월일 기재)   |  |  |  |  | | --- | --- | --- | --- | | 근 무 기 관 명 | 직 위 | 담 당 업 무 |
| --- | --- | --- | --- | --- | --- | --- | --- |
| 2008.3~ | 서울대병원 | 기금조교수 | 임상진료 |
| 2005.5 ~2008.2 | 서울대병원 | 임상교수 | 임상진료 |
| 2004.5∼2005.4 | 서울대병원 | 촉탁의 | 임상진료 |
| 2002.5∼2003.4 | 서울대병원 | 전임의 | 임상진료 |
| 1999.5∼2002.4 | 서귀포성심병원 | 과장 | 임상진료 |
| 1995.3∼1999.2 | 서울대병원 | 전공의 |  |
| 1994.3∼1995.2 | 서울대병원 | 인턴 |  |

최근 발표 논문

| 구분 | 제목 | 발표년월 | 발표 학술지 | | 저자  확인조서 | 분류 |
| --- | --- | --- | --- | --- | --- | --- |
|  |  |  | 학술지명 | 학술지  등재구분 |  |  |
| 정기  학술지 | Pulse pressure variation predict fluid responsiveness during heart displacement for coronary artery bypass graft surgery | 2011. 9 | J Cardiothorac Vasc Anesth | SCI | 교신 | 원저 |
| 정기  학술지 | Anesthetic management of a patient with Mounier-Kuhn  syndrome undergoing off-pump coronary artery bypass graft surgery-A case report | 2011.7 | Korean J Anesthesiolg | 학진등재지 | 교신 | 증례 |
| 정기  학술지 | Preoperative aspirin resistance does not increase myocardial injury during off-pump coronary artery bypass surgery | 2011.8 | J Korean Med Sci | SCIE | 교신 | 원저 |
| 정기  학술지 | Pulse pressure variation as a predictor of fluid responsiveness during one-lung ventilation for lung surgery using throarcotomy: randomised controlled study | 2011.1 | Eur J Anaesthesiol | SCIE | 교신 | 원저 |
| 정기  학술지 | The head-down tilt position decreases vasopressor requirement during hypotension following induction of anaesthesia in patients undergoing elective coronary artery bypass graft and valvular heart surgeries | 2011.1 | Eur J Anaesthesiol | SCIE | 교신 | 원저 |
| 정기  학술지 | The effect of remote ischaemic preconditioning on myocardial injury in pateitns undergoing off-pump coronary artery bypass graft surgery | 2010. 9 | Anaesth Intensive Care | SCIE | 교신 | 원저 |
| 정기  학술지 | Preoperative platelet resonse to collagen is associated with myocardial injury after off-pump coronary bypass graft in patients taking aspirin. | 2010. 2 | Korean J Anesthesiol | 학진등재시 | 교신 | 원저 |
| 정기  학술지 | Comparison of the bedside central venous catheter placement techniques: landmark vs electocardiogram guidance. | 2009. 5 | British J Anaesth | SCI | 공저자 | 원저 |
| 정기  학술지 | Comparison of thoracic epidural pressure in the sitting and lateral decubitus positions | 2008. 7 | Anesthesiology | SCI | 교신 | 원저 |
| 정기  학술지 | Effect of vasopressin on survival of Purkinje neurons in rat cerebellar slices after an in vitro simulated ischemia | 2009. 2 | Korean J Anesthesiol | 학진등재지 | 제1저자 | 원저 |
| 정기  학술지 | Perioperative cerebral infarct during cardiac surgery and changes in jugular venous O2 saturation and cerebral oximetry using near-infrared spectroscopy- A case report - | 2009.1 | Korean J Anesthesiol | 학진등재지 | 교신 | 증례 |
| 정기  학술지 | Left ventricular rupture immediately after mitral valve replacement-A case report | 2008.3 | Korean J Anesthesiol | 학진등재지 | 공저자 | 증례 |
| 정기  학술지 | Use of an extracorporeal membrane oxygenation device during bilateral sequential lung transplantation-A case report | 2008.8 | Korean J Anesthesiol | 학진등재지 | 공저자 | 증례 |
| 정기  학술지 | Anaphylaxis by atracurium on a cardiac surgery patient- A case report- | 2008.8 | Korean J Anesthesiol | 학진등재지 | 교신 | 증례 |
| 저서 | 마취과학III- 장기 이식 마취 (chapter) | 2009.11 |  |  | 공저 | 저서 |
