# Supplementary material for: Effect of Prewarming during Induction of Anesthesia on Microvascular Reactivity in Patients Undergoing Off-Pump Coronary Artery Bypass Surgery: A Randomized Clinical Trial
Source: PLoS One. 2016 Jul 21;11(7):e0159772. doi: 10.1371/journal.pone.0159772 (PMC4956040; doi:10.1371/journal.pone.0159772)
Supplement: S4 File — (DOCX) [file pone.0159772.s005.docx]

**Study Protocol**

**Inclusion criteria**

- Patients scheduled for OPCAB surgery at Seoul National University Hospital

- Age 20 - 85 years

**Exclusion criteria**

- Consent withdrawal

- Presence of anatomical anomaly or arteriovenous fistula in the upper extremities, diabetes requiring insulin therapy

- Peripheral vascular disease or recent cerebrovascular event (within 6 months)

- Preoperative left ventricular (LV) ejection fraction (EF) < 35%

- Requirement of preoperative inotropes or ventricular assist devices, redo or combined surgery, other than a conventional operative procedure (such as a minimally invasive approach)

- Pregnancy

**Randomization**

Control group, 20 patients

Prewarming group, 20 patients

Patients were randomized 1:1 to the prewarming or control group.

Block randomization (blocks of 4) was executed using a computer-generated program by an independent clinician and allocation concealment was kept until data analyses.

**Power analysis**

The primary endpoint was difference in recovery slope during vascular occlusion test (VOT) at 3 h after induction of anesthesia. We calculated the number of patients using the data from the heart surgery registry at Seoul National University Hospital: decrease in recovery slope at 3 h after induction of anesthesia was 1.52 ± 0.65 %/s (mean ± SD). From the results, we calculated the number of patients required to be 20 per group to detect a 50% decrease in recovery slope at 3 h after induction, relative to baseline, including a 10% dropout rate, with a two-sided design at a significance level of 5% and 90% power.

**Study protocol**

Before induction of anesthesia, T_core_ was measured at the tympanic membrane (T_tymp_) using a tympanic thermometer. The right and left T_tymp_ were measured, both twice, and the average of the measured temperatures was recorded. Two skin surface temperatures were monitored with skin temperature probes placed at the tip of the index finger and on the forearm. From them, temperature of finger (T_finger_) and forearm (T_forearm_) were monitored.

StO_2_ and StO_2_-derived parameters during VOT were monitored with an Inspectra™ StO_2_ Tissue Oxygenation Monitor (model 650; Hutchinson Technology Inc., Hutchinson, MN, USA) with an Inspectra™ StO_2_ Sensor (model 1615; Hutchinson Technology Inc.) attached to the thenar eminence of the hand of the opposite side for monitoring of T_finger_ and T_forearm_. VOT was performed by inflating the pneumatic cuff placed at the upper arm up to 50 mmHg above the systolic BP. Stable StO_2_ before each test was confirmed that changes in StO_2_ within 2% during 30 s. The cuff remained inflated until the StO_2_ reached 40% (occlusion of vessel), and was then deflated rapidly within 0.5 s. In case of inappropriate test, re-test was done after 5 min.

Patients in the prewarming group were heated using a forced-air warmer (Bair Hugger™ Model 505; 3M, St. Paul, MN, USA) connected to a full body blanket, also covered with a cotton blanket, with the air heating temperature set at 43°C. Patients were covered from the upper trunk to the lower leg except an arm for monitoring of skin temperatures. Prewarming was performed, after the placement of monitoring devices, until the end of induction of anesthesia. Patients in the control group were covered with a single cotton blanket without additional air heating.

Without premedication, patients were monitored with five-lead electrocardiography, pulse oximetry, non-invasive blood pressure (BP), bispectral index, and cerebral oximetry using a non-infrared spectroscopic sensor in the operating room. After local analgesia with 1% lidocaine injection, the right or left radial artery was cannulated with a 20-G Angiocath™ (Becton Dickinson Medical Ltd., Tuas, Singapore) and connected to a FloTrac™ transducer and EV1000™ monitor (Edwards Lifesciences LLC, Irvine, CA, USA). From them, arterial BP, cardiac index (CI), and stroke volume variation were monitored continuously. After placement of a central venous catheter (AVA HF; Edwards Lifesciences LLC) in the right or left internal jugular vein, a pulmonary artery (PA) catheter (Swan-Ganz CCOmbo V, model 774HF75; Edwards Lifesciences LLC) was inserted through the internal jugular lumen and was connected to a Vigilance™ II monitor (Edwards Lifesciences LLC) with *in vivo* calibration. CI and mixed venous saturation (SvO_2_) were monitored through the Vigilance™ II monitor after placing the PA catheter.

To treat intraoperative hypotension, intravenous inotropes, such as phenylephrine, ephedrine, vasopressin, calcium chloride, or continuous infusion of dobutamine, norepinephrine, or nitroglycerin were used and recorded. Anesthesia was maintained with continuous infusion of propofol (Fresofol®2 MCT 2%; Fresenius Kabi, Graz, Austria) and remifentanil (Ultiva™; GlaxoSmithKline, San Polo di-Torrile, Italy) in an effect site target-controlled infusion (TCI) mode. For TCI, the effect site concentration (Ce) was set to 1–4 μg/mL for propofol and 5–12 ng/mL for remifentanil using a commercial infusion pump. Anesthetic depth was monitored using an electroencephalogram-based monitor (BIS VISTA™ monitor; Aspect Medical Systems, Norwood, MA, USA). Vecuronium (0.04–0.08 mg/kg/h) was used for neuromuscular blockade. Crystalloid was used for fluid maintenance and blood products were transfused according to the hemodynamic and laboratory variables and to maintain hematocrit 26–30%.

All temperatures were recorded before the induction of anesthesia, and at 1, 2, and 3 h after induction of anesthesia, and at the end of surgery. Arterial blood gas analysis and blood test were performed at the same time points as the temperature recordings.

**Statistical analyses**

Analyses were performed using SPSS for Windows software (ver. 21.0.0.0; IBM, Armonk, NY, USA). Non-continuous variables, such as sex, placement of an arterial cannula, presence of co-morbidities, or smoking histories, were compared using χ^2^ test or Fisher’s exact test. Continuous variables, such as height, weight, body mass indices, hemodynamic microcirculatory parameters, or cardiac indices, were compared using Student’s *t*-test, the Mann-Whitney test. Serial changes in values were analyzed using repeated measures ANOVA or generalized estimating equation. A *p* value < 0.05 was taken to indicate statistical significance.
